# An Electrochemically‐Driven Reconstruction Strategy to Realize Highly Crystalline Covalent Organic Frameworks for Enhanced Hydrogen Evolution Reaction

**DOI:** 10.1002/advs.202501442

**Published:** 2025-03-19

**Authors:** Yuxin Ren, Shuang Li, Meidi Wang, Xue‐Qian Wu, Ya‐Pan Wu, Bojing Sun, Jun Zhao, Fangyuan Kang, Qichun Zhang, Dong‐Sheng Li

**Affiliations:** ^1^ College of Materials and Chemical Engineering Key Laboratory of Inorganic Nonmetallic Crystalline and Energy Conversion Materials China Three Gorges University Yichang Hubei 443002 P. R. China; ^2^ Hubei Three Gorges Laboratory Yichang Hubei 443007 P. R. China; ^3^ Department of Materials Science and Engineering City University of Hong Kong Tat Chee Avenue 83 Kowloon Hong Kong SAR 999077 P. R. China

**Keywords:** covalent organic frameworks, electrochemically driven reconstruction, high‐crystallinity, hydrogen evolution reaction

## Abstract

Developing diverse methods to approach highly crystalline covalent organic frameworks (COFs) for improvement of their electrocatalytic hydrogen evolution reaction (HER) activity is important but very challenging. Herein, for the first time, an electrochemically‐driven reconstruction strategy is demonstrated to convert semi‐polymerized low‐crystalline COFs into highly crystalline, structurally ordered COFs with enhanced HER activity. In situ and ex situ characterizations reveal that cyclic voltammetry (CV) cycles can promote crystallinity, thereby leading to improved conductivity, increased active site density, and superior stability. As a result, the highly crystalline COF achieves low overpotentials of 103.6 and 219.4 mV at 10 and 50 mA cm^−2^, respectively, with excellent stability (1200 h at 50 mA cm^−2^). More importantly, this strategy is generalizable and effective for various imine‐linked COFs with different bonding types, significantly improving their crystallinity and HER activity. This work not only establishes a novel method for constructing highly crystalline COFs but also demonstrates the versatility of electrochemically driven structural modulation in enhancing the catalytic performance of COFs.

## Graphical abstract

An electrochemically‐driven reconstruction strategy is proposed to convert low‐crystalline imine‐linked polymers into highly crystalline, structurally ordered COFs with enhanced HER activity, which offers new insights into the structural modulation of COFs and facilitates the design of more advanced metal‐free electrocatalysts.

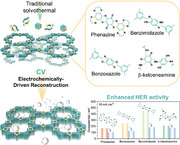

## 1 Introduction

With the increasing energy crisis and environmental problems, tremendous efforts have been made to explore carbon‐free and renewable energy sources.^[^ ^1^ ^]^ Fortunately, hydrogen is considered a promising clean energy source because it can meet the requirements of high energy density, abundant reserves, and zero carbon emissions.^[^ ^2^ ^]^ As a sustainable way to produce high‐purity hydrogen, electrochemical water splitting has drawn considerable attention.^[^ ^3^ ^]^ However, the cathodic hydrogen evolution reaction (HER) is often accompanied by a high overpotential owing to its slow kinetics, which limits the efficiency of hydrogen.^[^ ^4^ ^]^ Therefore, finding efficient electrocatalysts to accelerate reaction kinetics and reduce overpotential is highly focused.^[^ ^5^ ^]^ Over the past few decades, various metal‐based materials have been developed as HER catalysts in acidic solutions, including precious metals,^[^ ^6^ ^]^ transition metal oxides,^[^ ^7^ ^]^ nitrides,^[^ ^8^ ^]^ sulfides,^[^ ^9^ ^]^ phosphide,^[^ ^10^ ^]^ etc. Among these, Pt‐based materials are still regarded as the most effective HER catalysts due to their exceptional catalytic activity and stability, however, the high cost of precious metals remains a significant barrier to their large‐scale application.^[^ ^11^ ^]^ On the other hand, although non‐noble metal‐based compounds have been demonstrated to show promising HER catalytic activity, many catalysts suffer from issues such as corrosion and phase transition during electrocatalysis, which obstruct their catalytic performance.^[^ ^12^ ^]^ Therefore, there is a compelling requirement to develop metal‐free electrocatalysts that offer high HER activity, minimal resource loss, and superior stability.

In recent years, covalent organic frameworks (COFs), constructed through the covalent connection of organic building blocks, have garnered significant attention in the fields of energy storage and conversion due to their inherent porosity and structural designability.^[^ ^13^ ^]^ Particularly, the unique structural features of COFs make them have great potential applications in HER.^[^ ^14^ ^]^ One key advantage is that the catalytic active sites can be targeted by predesigning the periodic skeleton, and their tunable electronic structure can further provide opportunities to optimize the catalytic performance of COFs. Moreover, the regular porous architecture of COFs facilitates efficient ion diffusion and mass transfer, as well as the accessibility of active sites.^[^ ^15^ ^]^ Therefore, COFs are anticipated to exhibit excellent HER performance. However, the application of pure COFs in HER is still in its early stages.^[^ ^16^ ^]^ For example, in 2017, the Pradhan group reported the first two‐dimensional SB‐PORPy COF containing porphyrin and pyrene units, which exhibited an overpotential of ≈375 mV at 5 mA cm^−2^ for HER and a cyclic stability of 500 cycles.^[^ ^17^ ^]^ Subsequently, several pure COFs have been designed, but all of them demonstrated overpotentials exceeding 150 mV.^[^ ^18^ ^]^ Obviously, the reported HER performances of COF electrocatalysts are still far from the requirements for practical applications. This limitation can be attributed to several factors, such as poor electronic conductivity, slow reaction kinetics, and a limited number of active sites during the HER process. To address these issues, some methods, such as incorporating metal ions/metal clusters^[^ ^19^ ^]^ or conductive materials like graphene oxide^[^ ^20^ ^]^ and Ti_3_C_2_T*_x_* ^[^ ^21^ ^]^ have been explored. However, the introduction of additional components complicates the synthesis process and hinders the precise identification and regulation of active sites.^[^ ^22^ ^]^ Even worse, the structure of COFs may be destroyed during the synthesis process, and the introduced metallic ions are susceptible to corrosion in acidic media, which poses a challenge to the practical application of these catalysts.^[^ ^23^ ^]^ In general, achieving pure COFs electrocatalysts with high HER activity and stability remains a challenge nowadays.

As is well known, highly crystalline COFs are ideal candidate materials for electrocatalysis.^[^ ^24^ ^]^ Their highly ordered crystal structures not only guarantee a uniform distribution of active sites but also impart excellent stability, thus ensuring superior catalytic efficiency and maintaining activity over long periods during electrochemical processes.^[^ ^25^ ^]^ Moreover, the pore structure of the highly crystalline COFs is abundant and well‐ordered, which provides an optimal microenvironment and favorable ion transport channel for the catalytic reaction. Additionally, these COFs exhibit excellent electrical conductivity due to the fast electron transport pathways formed within their structures.^[^ ^26^ ^]^ This property facilitates the efficient transfer of electrons from the electrode surface to the catalytic active center, which in turn promotes the catalytic reaction. Nevertheless, in the synthesis of COFs, there is a trade‐off between high crystallinity and stability.^[^ ^27^ ^]^ On the one hand, the pursuit of highly crystalline COFs is often accompanied by the instability of reversible covalent bonds, which can easily decompose under harsh conditions, limiting their practical application. Conversely, employing irreversible covalent bonding to enhance stability usually results in low crystallinity, which affects the catalytic performance of COFs.^[^ ^28^ ^]^ A promising strategy to overcome this issue involves pre‐organizing monomers before polymerization. For instance, the Cooper group proposed a reconstruction strategy for COF synthesis, utilizing reversible and mobile covalent chains to pre‐organize monomers before irreversible polymerization.^[^ ^29^ ^]^ Inspired by this as well as the studies that have demonstrated structural evolution in inorganic electrocatalysts after electrochemical cycling,^[^ ^30^ ^]^ we hypothesize that semi‐polymerized low‐crystallinity COFs could be reconstructed into high‐crystallinity COFs under electrochemical driving, potentially leading to significant improvements in HER performance.

**Scheme 1:**
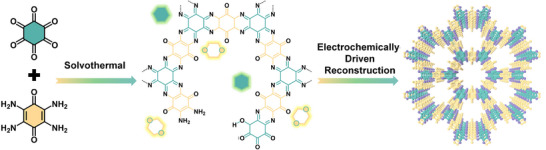
The schematic diagram for the synthesis of highly crystalline COF.

With these in mind, for the first time, we propose a dynamic electrochemically‐driven reconstruction strategy based on cyclic voltammetry (CV) cycles to achieve functional COFs with high crystallinity and stability, hereafter referred to as ED‐COF. As shown in **Scheme** **1**, using cyclohexanehexone and tetramine‐benzoquinone monomers as examples, we first synthesized a semi‐polymerized low‐crystallinity COF containing incomplete monomers via a conventional solvothermal method. Subsequently, we applied continuous CV cycles to transform the low‐crystalline COF into a highly crystalline structure with abundant C═N active sites, which serves as an efficient and robust HER electrocatalyst in acidic media. XRD analysis demonstrates that the crystallinity of the COF can be tuned by controlling the number of CV cycles. Notably, after 1000 CV cycles, the crystallinity reaches an optimal and stable level. Additionally, multiple characterizations reveal the formation of abundant C═N bonds and *π–π* ^*^ conjugation after CV cycles, further confirming the improved crystallinity of the COF following the electrochemical‐driven reconstruction. Compared with the initial COF, the ED‐COF exhibits a highly conjugated hexagonal planar structure with a uniform and dense distribution of C═N bonds, which ensures excellent chemical stability and provides enough catalytic active sites. Furthermore, the fully developed intra‐layer π‐electron conjugation system guarantees high electrical conductivity. Benefiting from these merits, the ED‐COF exhibits overpotentials of 103.6 and 219.4 mV at a current density of 10 and 50 mA cm^−2^, respectively, which are much smaller than those of initial COF (169.4 and 554.9 mV, respectively). Furthermore, the ED‐COF shows excellent HER stability, maintaining stable performance for 1200 h at the current density of 50 mA cm^−2^. These performance metrics far exceed those of pure COF electrocatalysts reported in the literature. Additionally, three other imine‐bonded COFs were synthesized, and the results show that they can also be transformed into highly crystalline COFs via the electrochemically‐driven reconstruction strategy, yielding enhanced HER performance. In short, this work pioneers the use of an electrochemically‐driven reconstruction strategy to efficiently and controllably synthesize highly crystalline COFs, offering a new pathway for the green and simple synthesis of functional COFs as well as their performance optimization.

## 2 Results and Discussion

**Figure 1:**
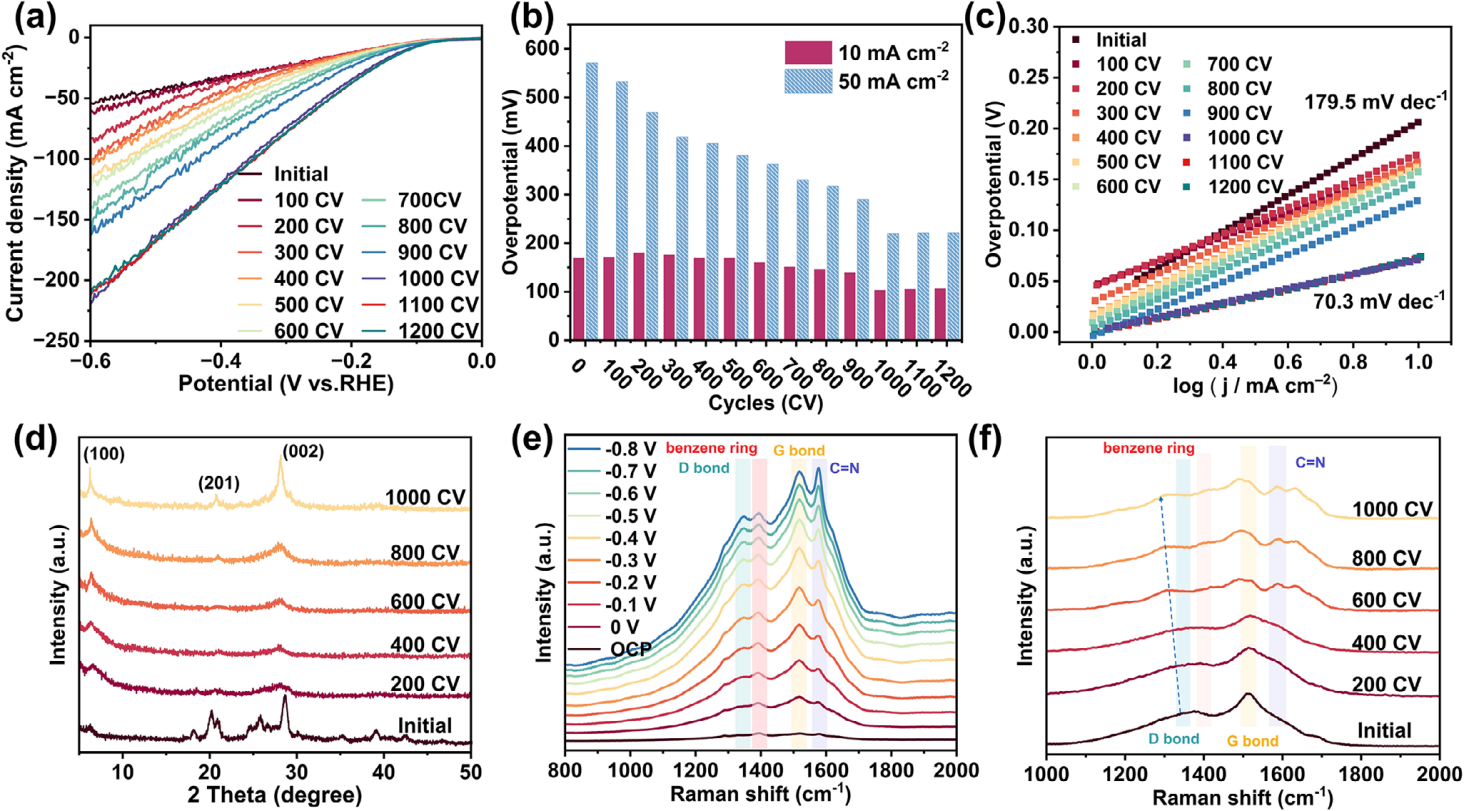
a) LSV curves of initial COF and after different CV cycles in 0.5 m H_2_SO_4_. Scan rate, 5 mV s^−1^. b) Corresponding overpotentials at 10 and 50 mA cm^−2^. c) Tafel plots. d) XRD patterns of initial COF and after different CV cycles. e) In situ Raman spectra of initial COF at various potentials. f) Raman spectra of initial COF and after different CV cycles.

We first conducted continuous CV cycles to investigate the effect of electrochemically‐driven reconstruction on the HER activity of initial COF. LSV curves were recorded after every 100 CV cycles. As shown in **Figure** **1**a, the current density of the initial COF gradually increases with the number of CV cycles, stabilizing after 1000 cycles. This suggests that the electrochemically‐driven reconstruction improves the HER activity of COF, with optimal performance achieved after 1000 cycles, which we designate as ED‐COF. Figure 1b shows the overpotentials of COF at current densities of 10 and 50 mA cm^−2^. As observed, the overpotential decreases progressively with increasing CV cycles. This trend is also reflected in the Tafel slope (Figure 1c), further indicating that electrochemically‐driven reconstruction enhances the charge transfer capability of COF to boost the HER activity. Additionally, the electrochemical double‐layer capacitance (*C*_dl_) was measured to assess the electrochemically active surface area of COF after varying CV cycles. As displayed in Figures S1,S2 (Supporting Information), the *C*_dl_ value escalates with the rising number of CV cycles, meaning an augmentation in the number of active sites within the COF under electrochemically‐driven reconstruction.

To explore the mechanism of the enhanced HER activity of COF through electrochemically‐driven reconstruction, we examined the phase evolution during the CV cycles using X‐ray diffraction (XRD). Figure 1d shows the XRD patterns of the initial COF and samples collected after every 200 CV cycles. As observed, the XRD pattern of initial COF presents two characteristic diffraction peaks at 6.3 ° and 28.3 °, which belong to the in‐plane reflection (100) and stacking reflection (002), respectively. The presence of the (201) peak at 20.7° verifies its AB‐stacking structure. However, except for these three characteristic peaks, some diffraction peaks corresponding to the monomers TABQ and CHHO were also observed (Figure S3, Supporting Information), confirming the existence of some unreacted monomers in the initial COF. After different CV cycles, the XRD patterns of the five samples only show the (100), (201), and (002) planes of the COF, suggesting that the products are high purity phase, revealing that the electrochemically‐driven reconstruction promotes the polymerization of the remaining monomers. Moreover, as the number of CV cycles increases, the diffraction peak intensity of COF intensifies, indicating an enhancement in its crystallinity after the electrochemically‐driven reconstruction. When 1000 CV cycles are applied, the crystallinity of the ED‐COF is the best, which can explain why the HER activity of COF reaches the optimal level after 1000 CV cycles. The microscopic morphology of the initial COF and ED‐COF was analyzed by scanning electron microscopy (SEM) and transmission electron microscopy (TEM). As shown in Figures S4–S6 (Supporting Information), the as‐obtained ED‐COF exhibits nanosheet morphology mirroring the initial COF, suggesting that the electrochemically‐driven reconstruction strategy does not alter the morphology of the COF. Figure S6b (Supporting Information) displays the high‐resolution HRTEM of ED‐COF with a lattice spacing of 0.235 nm, which corresponds to the (201) plane of COF. Besides, the elemental mapping images (Figure S6c, Supporting Information) confirm the co‐existence and uniform distribution of C, N, and O across the entire nanosheets of ED‐COF. The solid‐state ^13^C NMR spectrum further confirms the structure of ED‐COF (Figure S7, Supporting Information). To go further, N_2_ adsorption‐desorption isotherm measurement was performed to investigate the specific surface area and pore distribution of initial COF and ED‐COF. As shown in Figure S8 (Supporting Information), the specific surface area of the initial COF is calculated to be 16.62 m^2^ g^−1^, and the maximum pore size is 1.22 nm. The pore size is slightly smaller than the theoretically calculated pore size (1.5 nm), which may be caused by the distribution of unreacted monomers in the COF pores. After the electrochemically‐driven reconstruction, the specific surface area of ED‐COF is 23.47 m^2^ g^−1^, and the maximum pore size is 1.52 nm. The increase in specific surface area and pore size further confirms the polymerization of the remaining monomer during CV cycles. Besides, Figure S9 (Supporting Information) shows the thermogravimetric analysis (TGA) curves of initial COF and ED‐COF, revealing a significant difference in their thermal stability. The initial COF completely decomposes at 640 °C, indicating poor thermal stability. In contrast, ED‐COF exhibits much higher stability, with a total weight loss of ≈30% at 800 °C. These results indicate that ED‐COF is more stable, with a more robust and intact structure.

To gain a deeper understanding of the structural changes during the CV cycle process, in situ and ex situ Raman were conducted. Figure 1e shows the in situ Raman spectra of initial COF over the entire potential test range, in which the characteristic peaks at ≈1357 and ≈1578 cm^−1^ belong to the D and G bands of carbon materials, respectively. The D band is assigned to the vibration of sp^3^ hybrid carbon atoms and represents the defect structures of carbon materials. The G band corresponds to the vibration of sp^2^ carbon atoms, indicating the degree of graphitization.^[^ ^31^ ^]^ The intensity ratio of D and G bands (I_G_/I_D_) of COF enhances significantly with increasing potential (Figure S10, Supporting Information), suggesting that the degree of graphitization is greatly improved, which further confirms the high crystallinity of COF under electrochemically‐driven reconstruction. Notably, there is a splitting phenomenon in both the D and G bands, which may be caused by the different coordination environments of carbon atoms in COF.^[^ ^32^ ^]^ In particular, the G band splitting becomes more pronounced as the potential increases, which could be attributed to the increase in interlayer coupling and the enhancement of ordered stacking of COF in the HER process.^[^ ^33^ ^]^ Ex‐situ Raman spectra (Figure 1f) also show that the D and G bands of COF split obviously with the increase of CV cycles, accompanied with the D‐band peak shifts to lower frequency, which further reveals the influence of electrochemically‐driven reconstruction on the structure of COF.

**Figure 2:**
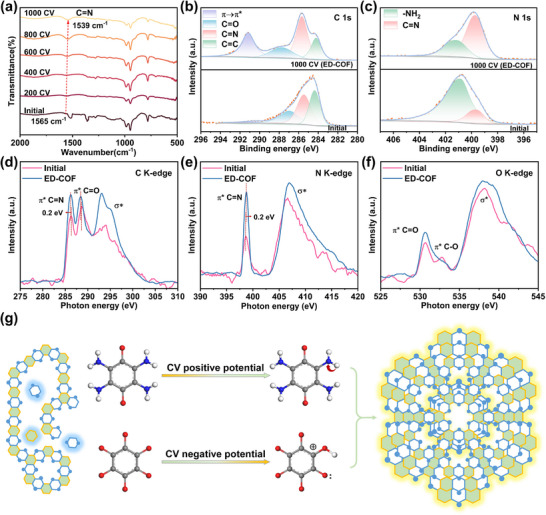
a) FT‐IR spectra of initial COF and after different CV cycles. XPS spectra of b) C 1s, c) N 1s, d) C K‐edge, e) N K‐edge, and f) O K‐edge of initial COF and ED‐COF. g) Mechanism diagram of CV electrochemically‐driven reconstruction.

Additionally, Fourier transform infrared spectroscopy (FT‐IR) was used to investigate the evolution of functional groups during the CV cycles. As shown in Figure S11 (Supporting Information), the peaks of initial COF at 1527 and 1358 cm^−1^ correspond to N‐H and C‐N stretching vibrations of unpolymerized monomers. After electrochemically‐driven reconstruction, the N‐H and C‐N peaks of initial COF almost disappear (**Figure** **2**a), suggesting that the polymerization degree is improved. Moreover, with the rising number of CV cycles, the peak position of C═N peaks belonging to the phenazine bond decreased from 1565 to 1539 cm^−1^, accompanied by an increase in peak intensity. This change signifies an increase in the conjugation of the COF during the electrochemically‐driven reconstruction, which aligns well with the high crystallinity observed in ED‐COF.

The surface elemental composition and chemical environment of initial COF and ED‐COF were analyzed by XPS. Figure S12 (Supporting Information) shows their survey XPS spectra, confirming the existence of C, N, and O elements. C 1s spectrum of initial COF (Figure 2b) is deconvoluted into four peaks at 284.2, 285.7, 287.8, and 291.2 eV, which are attributed to C═C, C═N, C═O, and *π–π* ^*^ conjugation, respectively.^[^ ^34^ ^]^ Compared with the initial COF, the peak area of C═N bond in ED‐COF is significantly enhanced, indicating the formation of more quinoxaline linkages within the COF. Additionally, the peak intensity of *π–π* ^*^ conjugation for ED‐COF is markedly increased, which further provides direct evidence of structural optimization in ED‐COF induced by electrochemically‐driven reconstruction. The *π–π* ^*^ conjugation contributes to the charge transfer rate of the COF, effectively improving its electrical conductivity.^[^ ^35^ ^]^ The formation of quinoxaline linkages is reflected more clearly in the N 1s spectrum. The finely scanned N 1s XPS spectrum of initial COF in Figure 2c shows two peaks at 399.8 and 400.9 eV, which are assigned to C═N and ‐NH_2_ bonds, respectively. After 1000 CV cycles, the relative peak area of the C═N bond in ED‐COF is remarkably larger than that of the initial COF. Moreover, from initial COF to ED‐COF, the peak area ratio of C═N to ─NH₂ rising from 0.87 to 1.08. This change is likely related to the protonation of the monomers induced by the local electric field in the electrochemical environment, which facilitates the transformation of ─NH_2_ bond into C═N double bond, thereby enhancing the conjugation feature and the number of catalytic active sites of COF.

**Figure 3:**
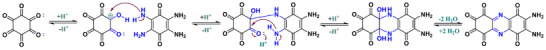
The model reaction steps of phenazine bond formation.

For a more profound understanding of the local electronic states of the initial COF and ED‐COF, soft X‐ray absorption near‐edge structure (XANES) characterization was conducted. As illustrated in Figure 2d, the C K‐edge spectra exhibit three distinct characteristic peaks at 286.2, 288.5, and 293.2 eV, corresponding to π^*^C═N, π^*^C═O, and σ^*^ anti‐bonding states, respectively.^[^ ^36^ ^]^ In comparison to the initial COF, the normalized peak intensity and area of the ED‐COF are greatly increased, further confirming that electrochemically‐driven reconstruction promotes monomer polymerization to generate more C═N bonds. Additionally, the peaks of ED‐COF shift toward lower photon energies relative to the initial COF, indicating an increased electronic density around C atoms after electrochemically driven reconstruction. This shift aligns with the enhanced conjugation resulting from the highly crystalline ED‐COF. For the N K‐edge spectrum (Figure 2e), two peaks of ED‐COF are identified at 398.8 and 406.9 eV, which belong to the 1s electronic transitions of N to π^*^C═N and σ anti‐bonding states, respectively.^[^ ^37^ ^]^ These peak intensity and area of ED‐COF are both increased compared to the initial COF, indicating a significant alteration in the local coordination environment of the N atoms after electrochemically‐driven reconstruction, providing further evidence for the formation of C═N bonds. Moreover, the shift of the C═N bond toward higher photon energy suggests a decrease in electron density around the N atoms. The shifted XANES peaks of C 1s and N 1s observed in ED‐COF indicate that a fraction of electrons may be transferred from N to C, which can be attributed to the high conjugation of ED‐COF. Additionally, in the O K‐edge spectrum of initial COF (Figure 2f), the resonance peaks at 530.7, 532.8, and 538.2 eV correspond to π^*^C═O, σ^*^C─O, and high‐energy transitions of oxygen atoms, respectively.^[^ ^38^ ^]^ Compared with the initial COF, except for the obvious increases in the peak position and area of ED‐COF, the disappearance of the σ^*^C─O resonance peak suggests that some excess exposed oxygen groups are effectively converted in the original COF, which is consistent with the re‐polymerization of monomers during the electrochemically‐driven reconstruction process. This change not only stabilizes the electronic structure of the oxygen groups but may also reduce the interference between the oxygen groups, thereby enhancing the overall order and catalytic activity of the COF.^[^ ^39^ ^]^ Based on these results, we hypothesized the mechanism underlying the improvement in crystallinity of COF as a result of electrochemically driven reconstruction. In detail, semi‐polymerized low‐crystalline COF (initial COF) shows significant flexibility in spatial volume and structural adjustment due to incomplete cross‐linking and crystallization processes. As displayed in Figure 2g, In the CV process, the variation of applied potential drives the redox reactions of amine (−NH_2_) and carbonyl (C═O) precursors, thereby facilitating the formation of imine bonds (C═N). As the potential increases, the ‐NH_2_ group is converted into amine cations (R‐NH_2_^+^), which enhances its nucleophilicity and facilitates its nucleophilic attack on the C═O group. Conversely, when the potential decreases, the C═O group is reduced to alcohol groups (‐CH_2_OH), which increases its electrophilicity and reactivity toward the ‐NH_2_ group. This modulation of potential effectively balances the nucleophilicity and electrophilicity of the reactants, thus efficiently promoting the formation of imine bonds. The basic chemical steps of phenazine bond formation, as illustrated in **Figure** **3**, involve three key steps: nucleophilic attack of the amine on the carbonyl group, rearrangement of the intermediate, and the release of a water molecule, ultimately leading to the formation of a stable phenazine bond (C═N). The electrochemically‐driven reconstruction enables the initial COF to undergo secondary polymerization and achieve a deeper level of structural optimization, thereby improving crystallinity. A high crystallinity results in an ordered molecular arrangement, which not only enhances the structural stability but also guarantees the uniform distribution of active sites. Moreover, as crystallinity increases, the π*–*π stacking becomes tighter, leading to the enhancement in electrical conductivity. In short, the ED‐COF with an optimized structure would have excellent catalytic activity and stability.

**Figure 4:**
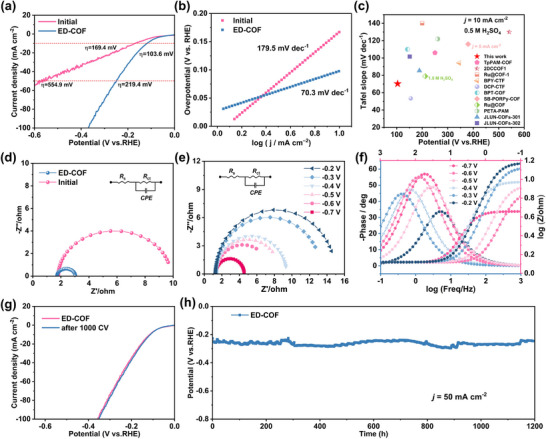
HER performance in acid media: a) LSV curves of initial COF and ED‐COF. b) Tafel plots of initial COF and ED‐COF. c) Comparison of HER activity of ED‐COF with other catalysts. d) EIS of initial COF and ED‐COF. e) Nyquist plots of ED‐COF under different voltages. f) Phase angle and Warburg plots of ED‐COF under different voltages. g) LSV curves of ED‐COF before and after 1000 CV cycles. h) Chronopotentiometry curve of ED‐COF for 1200 h at 50 mA cm^−2^.

To validate the hypothesis, the HER performance of ED‐COF was thoroughly assessed using a standard three‐electrode system in 0.5 H_2_SO_4_. As shown in the polarization curves (**Figure** **4**a), the HER performance of ED‐COF is significantly superior to that of the initial COF. It only requires an overpotential of 103.6 and 219.4 mV to reach a current density of 10 and 50 mA cm^−2^, respectively, which are much lower than those of initial COF (169.4 and 554.9 mV, respectively). This remarkable enhancement of HER activity can be attributed to the increased crystallinity in COF. Tafel slope is a critical parameter to evaluate the HER kinetics of electrodes. As shown in Figure 4b, ED‐COF exhibits a Tafel slope of 70.3 mV dec^−1^, substantially lower than that of the initial COF (179.5 mV dec^−1^), indicating faster kinetics for the HER process. Moreover, the low overpotential and Tafel slope of ED‐COF are superior to the vast majority of previously reported COF‐based HER catalysts (Figure 4c; Table S1, Supporting Information), highlighting the advantages of electrochemically‐driven reconstruction for the structural optimization of COFs. To further investigate the reaction kinetics and hydrogen adsorption behavior on ED‐COF during the HER process, electrochemical impedance spectroscopy (EIS) was conducted. The Nyquist plots simulated using the equivalent circuit (inset in Figure 4d) reveal that the semicircular diameter of ED‐COF is significantly smaller compared to that of the initial COF, indicating a more efficient electron transfer process. Furthermore, the Nyquist plots of ED‐COF under different potentials (Figure 4e) demonstrate a progressive reduction in the semicircular diameter with increasing potential, suggesting accelerated charge transfer and a decreased adsorption strength of hydrogen atoms at intermediate states on the electrode surface.^[^ ^40^ ^]^ As a complementary representation of EIS, the Bode plots provide deeper insights into the catalytic kinetics of the electrode. As shown in Figure 4f, the curves on the left illustrate the correlation between phase angle and frequency, featuring a prominent peak in the low‐frequency region, which reflects the relatively facile electron transfer process at the electrode‐electrolyte interface. When the potential increases from −0.2 to −0.7 V, the phase angle gradually increases from 33.6 ° to 56.9 °, indicating that the charge transfer process becomes more efficient and the reaction rate gradually improves. The right curves show the relationship between impedance amplitude and frequency. As the potential increases from −0.2 to −0.7 V, both the slope and the termination value of the Log Z gradually decrease from 1.2 to 0.7, reflecting an enhancement in the electron transfer rate, thereby accelerating the HER process.^[^ ^41^ ^]^ Stability is another critical parameter to evaluate the practical performance of catalysts. Therefore, continuous CV cycling and long‐term chronopotentiometry measurements were performed to study the durability of ED‐COF in acid media. As displayed in Figure 4g, the LSV curves after 1000 cycles exhibit negligible differences, indicating the excellent catalytic stability of ED‐COF during the HER process. Furthermore, even after 1200 h of continuous operation at a constant current density of 50 mA cm^−2^, the chronopotentiometry curve of ED‐COF shows only a slight potential change (Figure 4h), providing further evidence of its outstanding durability for HER. The post‐HER characterizations of the ED‐COF, including XRD, TEM, XPS, and UV–vis were performed after the stability test. As displayed in Figures S13–S16 (Supporting Information), the phase, morphology, chemical state, and composition of ED‐COF are maintained well after the long‐term HER process, further revealing the excellent structural stability in acid media. All the above results highlight the ED‐COF is an efficient and stable electrocatalyst for HER.

**Figure 5:**
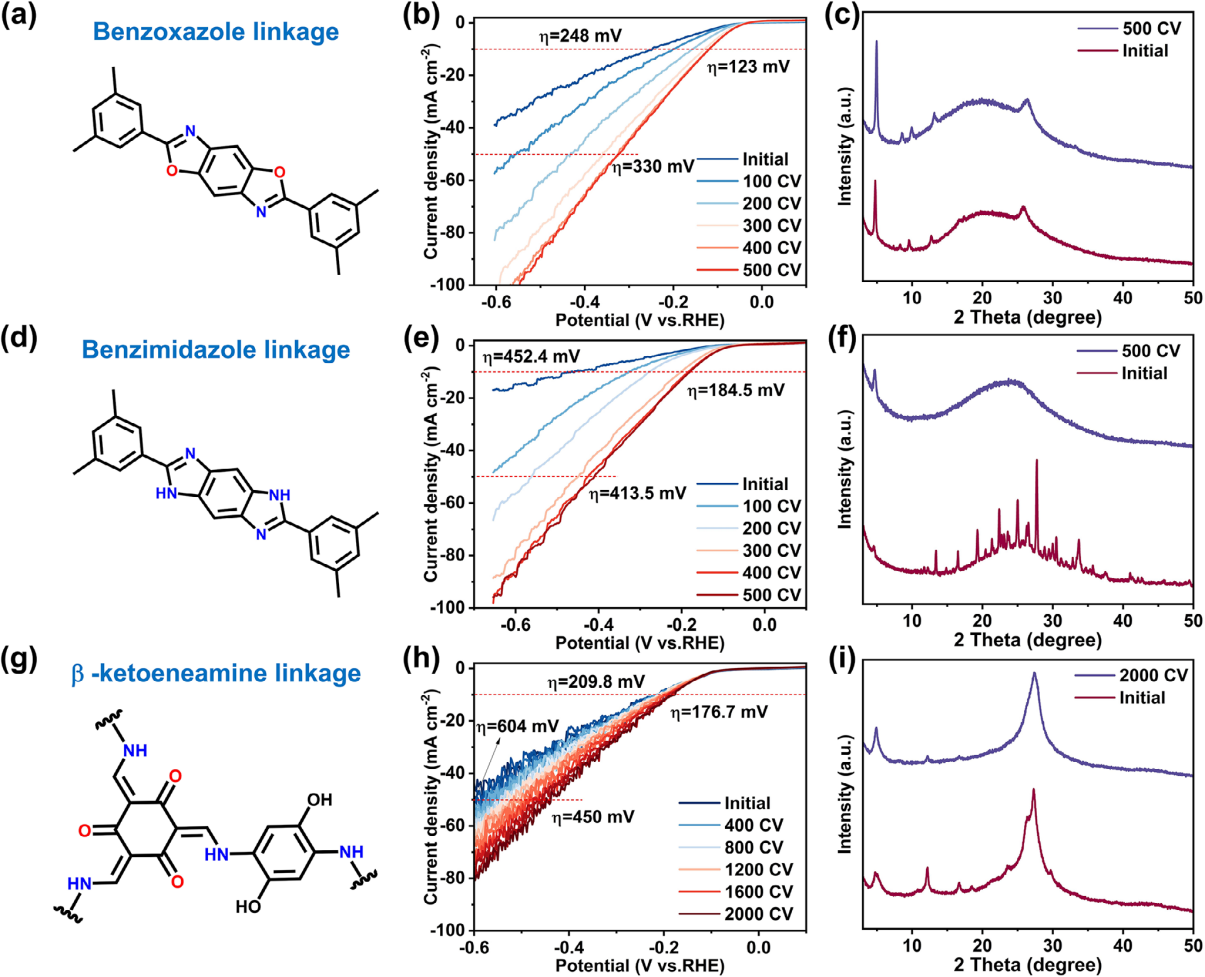
Electrochemically‐driven reconstruction for the synthesis of other imine‐bonded COFs: a) Benzoxazole linkage COF, b) its LSV curves initial and after different CV cycles, c) Comparison of XRD patterns of initial COF and after 500 CV. d) Benzimidazole linkage COF, e) its LSV curves initial and after different CV cycles, f) Comparison of XRD patterns of initial COF and after 500 CV. g) β‐ketoeneamine linkage COF, h) its LSV curves initial and after different CV cycles, i) Comparison of XRD patterns of initial COF and after 2000 CV.

To verify the universality of the effect of electrochemically‐driven reconstruction strategy on the crystallinity of COF, we conducted the same tests on three other typical imine‐bonded COFs: benzoxazole linkage (**Figure** **5**a), benzimidazole linkage (Figure 5d), and *β*‐ketoenamine linkage (Figure 5g) COFs. As shown in Figure 5b,e,h, the HER activity of all samples is significantly enhanced after electrochemically‐driven reconstruction. Meanwhile, XRD (Figure 5c,f,i) and XPS (Figures S17–S19, Supporting Information) analyses confirm an improvement in their crystallinity. Specifically, when the number of CV cycles is 500, the HER activity of benzoxazole linkage COF and benzimidazole linkage COF is optimal and stable. The overpotentials at 10 and 50 mA cm^−2^ of benzoxazole linkage COF (Figure 5b) decrease from 248 and 580 mV to 123 and 330 mV, respectively. Similarly, the benzimidazole linkage COF (Figure 5e) shows a decrease in overpotential from 452.4 to 184.5 mV at 10 mA cm^−2^. For β‐ketoenamine linkage COF (Figure 5h), the overpotentials at 10 and 50 mA cm^−2^ of the original COF have reduced from 209.8 and 604 mV to 176.7 and 450 mV, respectively. Notably, the XRD pattern of benzimidazole linkage COF (Figure 5f) reveals that the initial COF contains many incomplete monomers. In the electrochemically‐driven reconstruction process, its structure has a greater capacity for tunability, and its crystallization undergoes a more pronounced improvement. As a result, the HER activity of benzimidazole linkage COF experiences a higher degree of enhancement. All the above results confirm that the electrochemically‐driven reconstruction strategy is generally applicable to different types of imine COF, which can effectively optimize the crystallinity of COF and significantly improve HER performance, particularly for COF with a relatively low initial degree of polymerization.

## 3 Conclusion

In summary, we have developed an electrochemically‐driven reconstruction strategy to successfully convert semi‐polymerized low‐crystallinity phenazine‐based COF into highly crystalline ED‐COF with outstanding stability. Through CV cycles, ED‐COF demonstrates a significant enhancement of HER performance, with low overpotential (103.6 mV at 10 mA cm^−2^) and excellent stability (1200 h at 50 mA cm^−2^), which notably outperforms conventional pure COF electrocatalysts. A series of ex‐situ and in situ characterizations reveal that the crystallinity of initial COF undergoes a corresponding enhancement with increasing CV cycles, accompanied by enhanced conjugation, improved stability, and an increased number of active sites, collectively contributing to an overall boosted HER performance. Moreover, this strategy demonstrates its universality for other imine‐bonded (C═N) COFs with different structures. Through this CV‐driven strategy, three other types of COFs can be obtained with enhanced crystallinity and catalytic activity. This work provides an efficient and controllable approach for the preparation of highly crystalline COFs and the optimization of their HER performance. The electrochemically‐driven reconstruction strategy holds the promise for extending to other COFs‐based electrochemical systems, thereby advancing the synthesis and catalysis applications of highly crystalline COFs.

## Conflict of Interest

The authors declare no conflict of interest.

## Supporting information



Supporting Information

## Data Availability

The data that support the findings of this study are available in the supplementary material of this article.
