# Supplementary material for: An Electrochemically‐Driven Reconstruction Strategy to Realize Highly Crystalline Covalent Organic Frameworks for Enhanced Hydrogen Evolution Reaction
Source: Adv Sci (Weinh). 2025 Mar 19;12(18):2501442. doi: 10.1002/advs.202501442 (PMC12079546; doi:10.1002/advs.202501442)
Supplement: Supplementary file 1 — Supporting Information [file ADVS-12-2501442-s001.docx]

**S1. Experimental Section**

**Synthesis of Initial TQBQ-COF.**

Tetramine-benzoquinone (TABQ, 50.4 mg, 0.3 mmol) and cyclohexanehexone (CHHO, 62.4 mg, 0.2 mmol) were firstly added into a Pyrex tube (10 mL), followed by the addition of deoxygenated NMP (3 mL) and sulfuric acid (>98%, 30 μL) into the same tube. Next, the mixture was sonicated for 10 min, and then, the tube was flash-frozen at 77 K (liquid N_2_ bath) and degassed through three freeze-pump-thaw cycles. Then, the tube was sealed under vacuum and heated at 65 °C for 20 h. The resulting product was collected by filtration and washed with water and methanol until the filtrate was colorless. The dark brown product was dried under vacuum at 60 °C for 24 h.


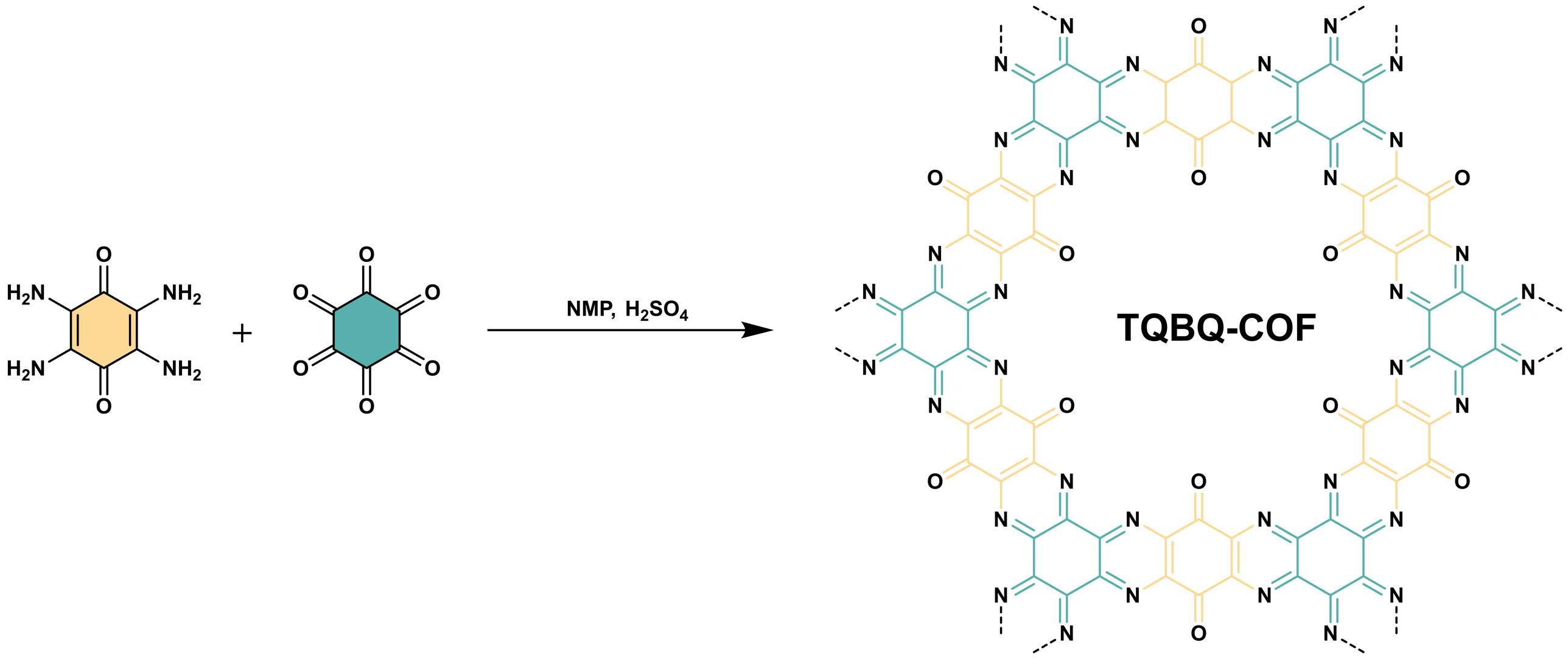


**Synthesis of Benzoxazole linkage COF (LZU-190).**

1,3,5-Triformylbenzene (TFB, 10.8 mg, 0.067 mmol), 2,5-Diaminohydroquinone dihydrochloride [Pa-(OH)_2_•2HCl, 21.3 mg, 0.10 mmol] and benzimidazole (35.4 mg, 0.30 mmol) were added into a Pyrex tube (10 mL), followed by the addition of deoxygenated NMP (0.45 mL) and mesitylene (0.45 mL) into the same tube. ^[1]^ Next, the mixture was sonicated for 10 min before the tube was flash-frozen at 77 K (liquid N_2_ bath) and degassed through three freeze-pump-thaw cycles. Then, the tube was sealed under vacuum and heated at 65 °C for 20 h. The resulting product was collected by filtration and washed with water and DMF until the filtrate was colorless. The brown product was dried under vacuum at 60 °C for 24 h.


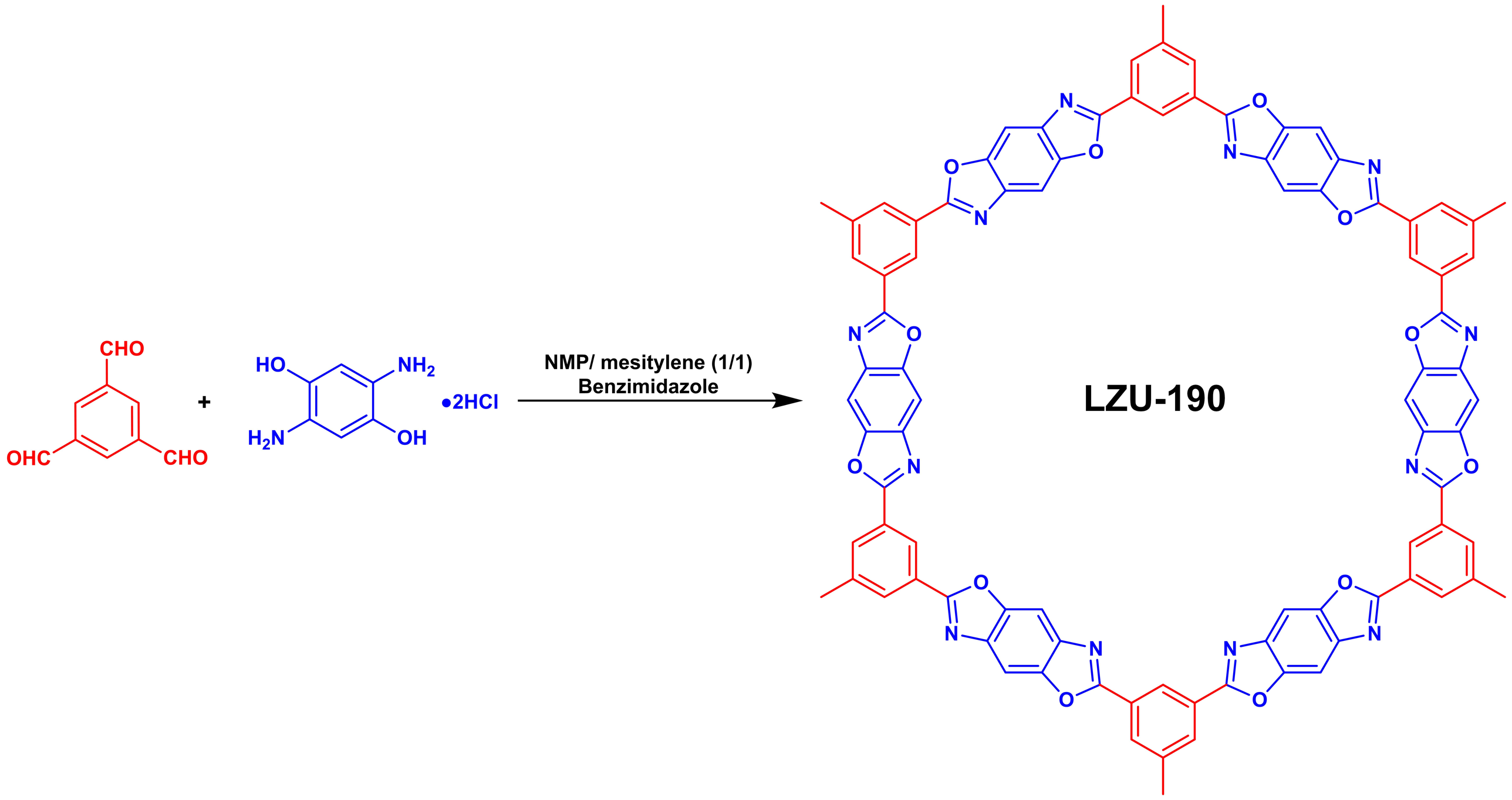


**Synthesis of Benzimidazole linkage COF (****TFB-TAB).**

1,3,5-Triformylbenzene (TFB, 12.3 mg, 0.076 mmol), 1,2,4,5-Benzenetetramine (TAB, 48.3 mg, 0.11 mmol) and imidazole (6.8 mg, 0.10 mmol) were added into a Pyrex tube (10 mL), followed by the addition of 4 mL mesitylene/NMP/dioxane (2:1:1) mixture into the same tube. ^[2]^ Next, the entire mixture was sonicated for 10 min, before the tube was flash-frozen at 77 K (liquid N_2_ bath) and degassed through three freeze-pump-thaw cycles. Then, the tube was sealed under vacuum and heated at 65 °C for 20 h. The resulting product was collected by filtration and washed several times with acetone. The black brown product was dried under vacuum at 60 °C for 24 h.


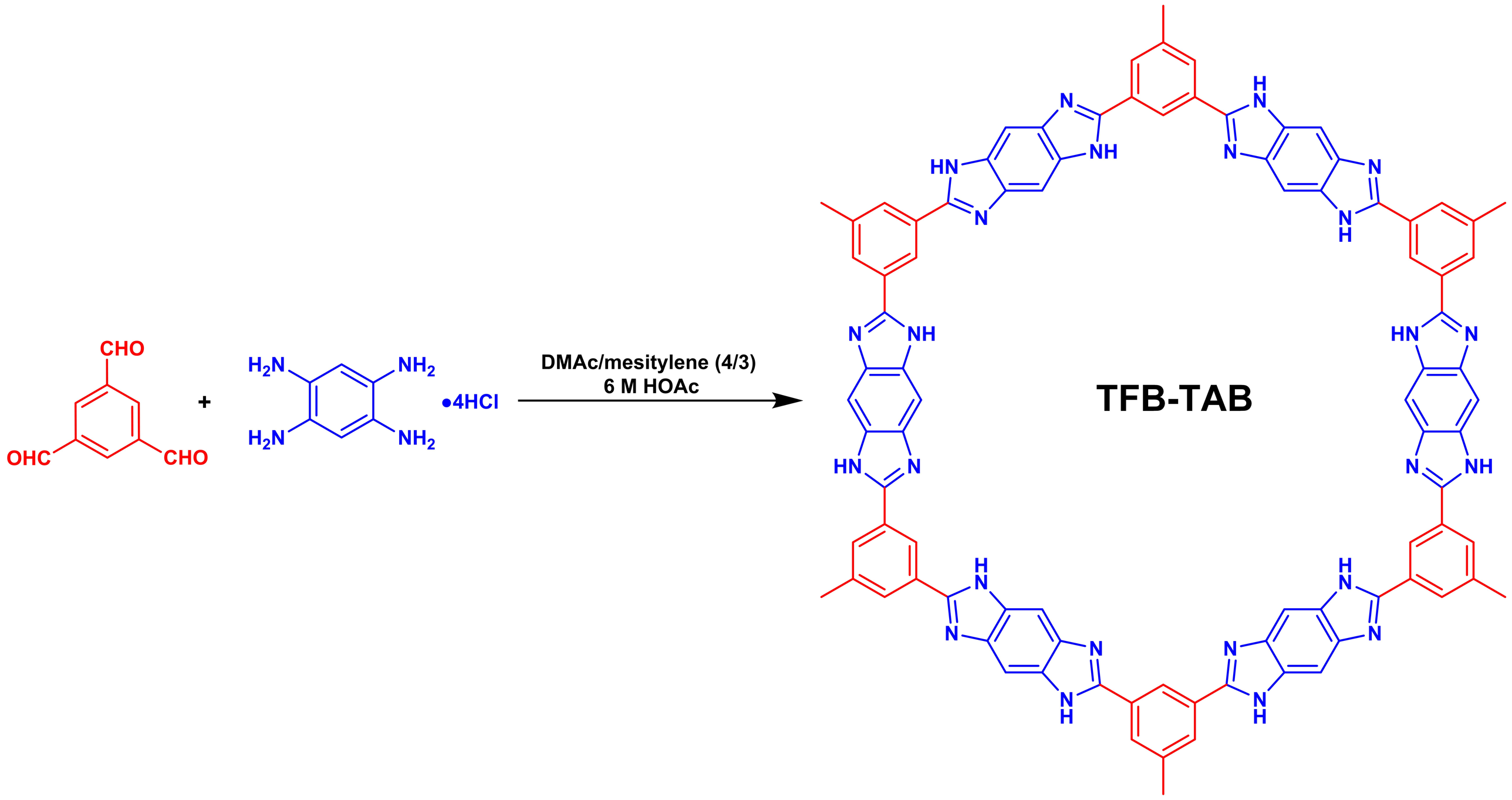


**Synthesis of β-ketoeneamine linkage COF (TpPa-(OH)_2_).**

1,3,5-Triformylphloroglucinol (TFp, 21.3 mg, 0.1 mmol) and 2,5-Diaminohydroquinone dihydrochloride [Pa-(OH)_2_•2HCl, 32.0 mg, 0.15 mmol] were introduced into a Pyrex tube (10 mL), followed by the addition of 3 mL (1:1) mixture of mesitylene and 1,4-dioxane, along with 0.5 mL of 6 M acetic acid, into the same tube.^[3]^ Next, the mixture was sonicated for 10 min before the tube was flash-frozen at 77 K (liquid N_2_ bath) and degassed through three freeze-pump-thaw cycles. Then, the tube was sealed under vacuum and heated at 65 °C for 20 h. The resulting product was collected by filtration and washed with DMAc followed by water and finally acetone for several times. The dark red product was dried under vacuum at 60 °C for 24 h.


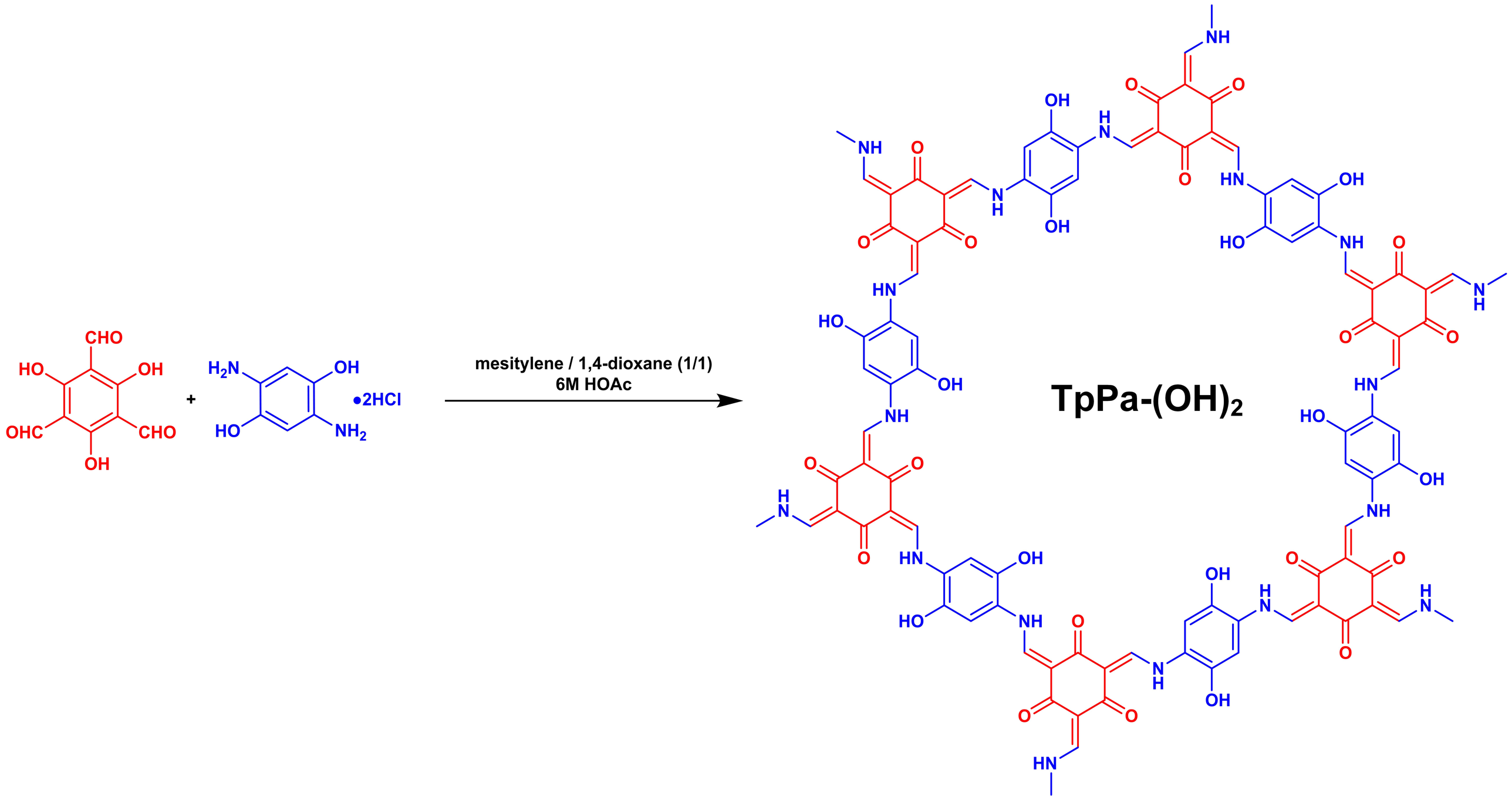


**Material Characterization**

The phase composition and crystallinity of the samples were analyzed by X-ray diffraction (XRD) using a Rigaku Ultima IV diffractometer with Cu Kα radiation (λ = 1.5406 Å). The microstructure of the sample was examined by scanning electron microscopy (SEM, JSM-7500F) and transmission electron microscopy (TEM, JEOL-F200), providing insights into the surface morphology and internal structure. Fourier transform infrared spectroscopy (FT-IR) analysis was performed on a PerkinElmer spectrometer to identify functional groups and chemical bonding within the material. Raman spectroscopy was conducted on a Thermo Scientific DXR laser confocal Raman spectrometer to investigate the vibrational modes and structural information of the sample. N₂ adsorption-desorption isotherms were measured on a Belsorp Max apparatus (MICROTRAC) to evaluate the specific surface area and pore size distribution, calculated by the BET and NLDFT methods. ¹³C NMR spectra were recorded on a Bruker AVANCE II HD 600 MHz spectrometer to provide detailed information on the carbon environment. The surface chemical composition and valence state of the sample were confirmed by X-ray photoelectron spectroscopy (XPS, ESCALAB250XI). Thermal properties were studied by thermogravimetric analysis (TGA) using a HENVEN-Automatic Differential Thermal Analyzer HQT-1 instrument to investigate the material's thermal stability and decomposition behavior. Ultraviolet–visible (UV-Vis) absorption spectra were recorded on a Shimadzu UV-3600 iPlus spectrophotometer to investigate the dissolution behavior of the material by examining its light absorption characteristics. The detailed electronic structure of the sample was further investigated by X-ray absorption near edge structure (XANES) at the BL12B beamline (MCD-A and MCD-B stations) of the National Synchrotron Radiation Laboratory (Hefei, China).

**Electrochemical measurements**

The electrochemical measurements were conducted on the CHI660E electrochemical workstation using a standard three electrode system. The as-obtained catalyst was coated on carbon cloth with a loading amount of 0.5 mg cm^-2^ as the working electrodes. The graphite rod and Ag/AgCl were used as the counter electrode and reference electrode, respectively. The linear sweep voltammetry (LSV) was measured at a scan rate of 5 mV s^-1^. Electrochemical active surface area was estimated using cyclic voltammetry (CV) in the potential range between 0.06 to 0.16 V with scan rates of 20, 40, 60, 80, 100 mV s^-1^. The electrochemical impedance spectroscopy (EIS) was conducted at -0.2, -0.3, -0.4, -0.5, -0.6, -0.7 V (vs. Ag/AgCl) in the frequency range of 100 to 0.1 Hz. All potentials were converted to the reversible hydrogen electrode (RHE) according to the formula: E_RHE_ = E_Ag/AgCl_ + E^θ^_Ag/AgCl_ + 0.059 pH (V). Moreover, all potentials without IR compensation.

**S2. Additional characterization information**

**

**

**Figure S1** CV curves at different scan rates of (a) Initial COF and (b-f) after different CV.

**
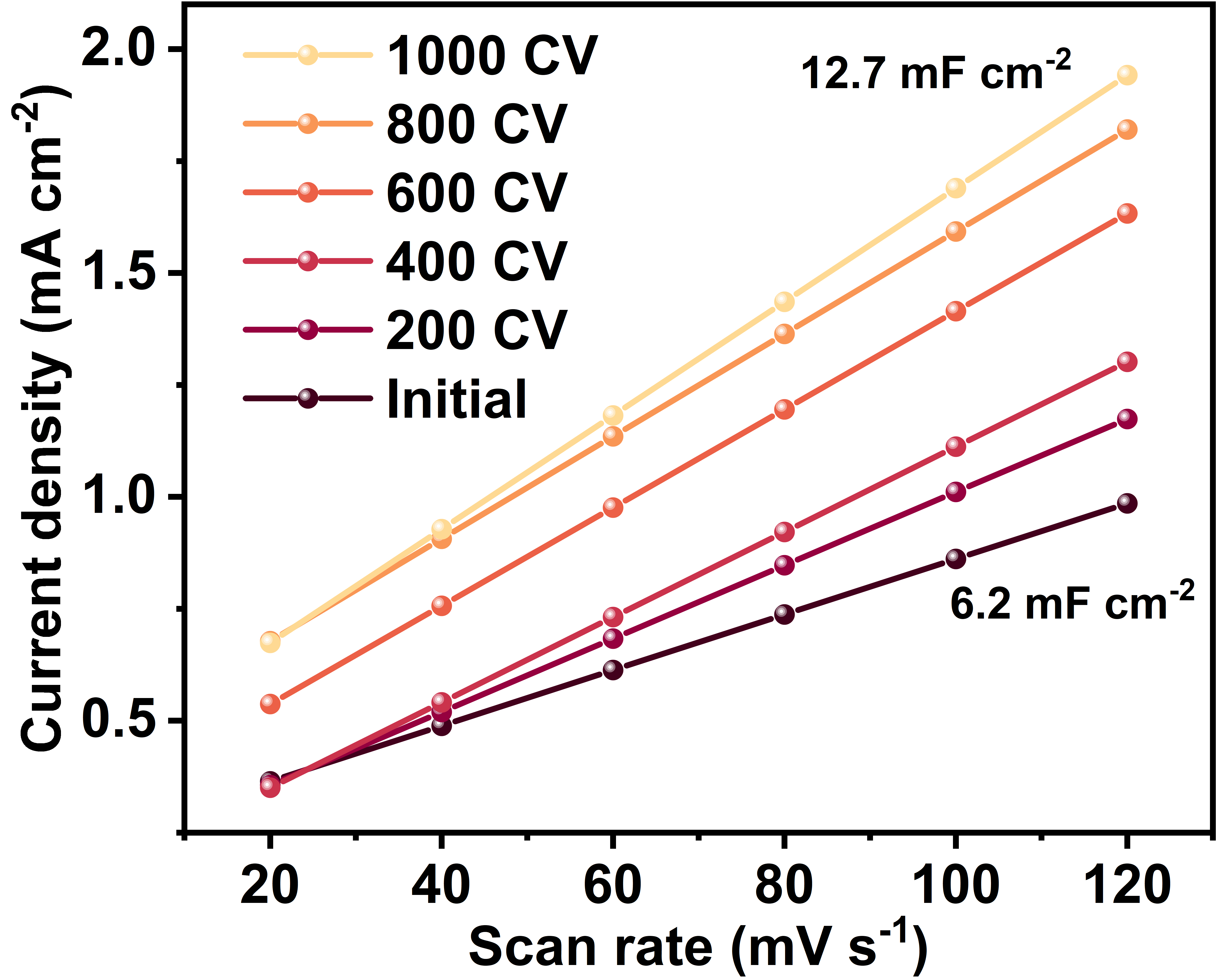
**

**Figure S2** The corresponding values of *C*_dl_ of initial COF and after different CV.

**
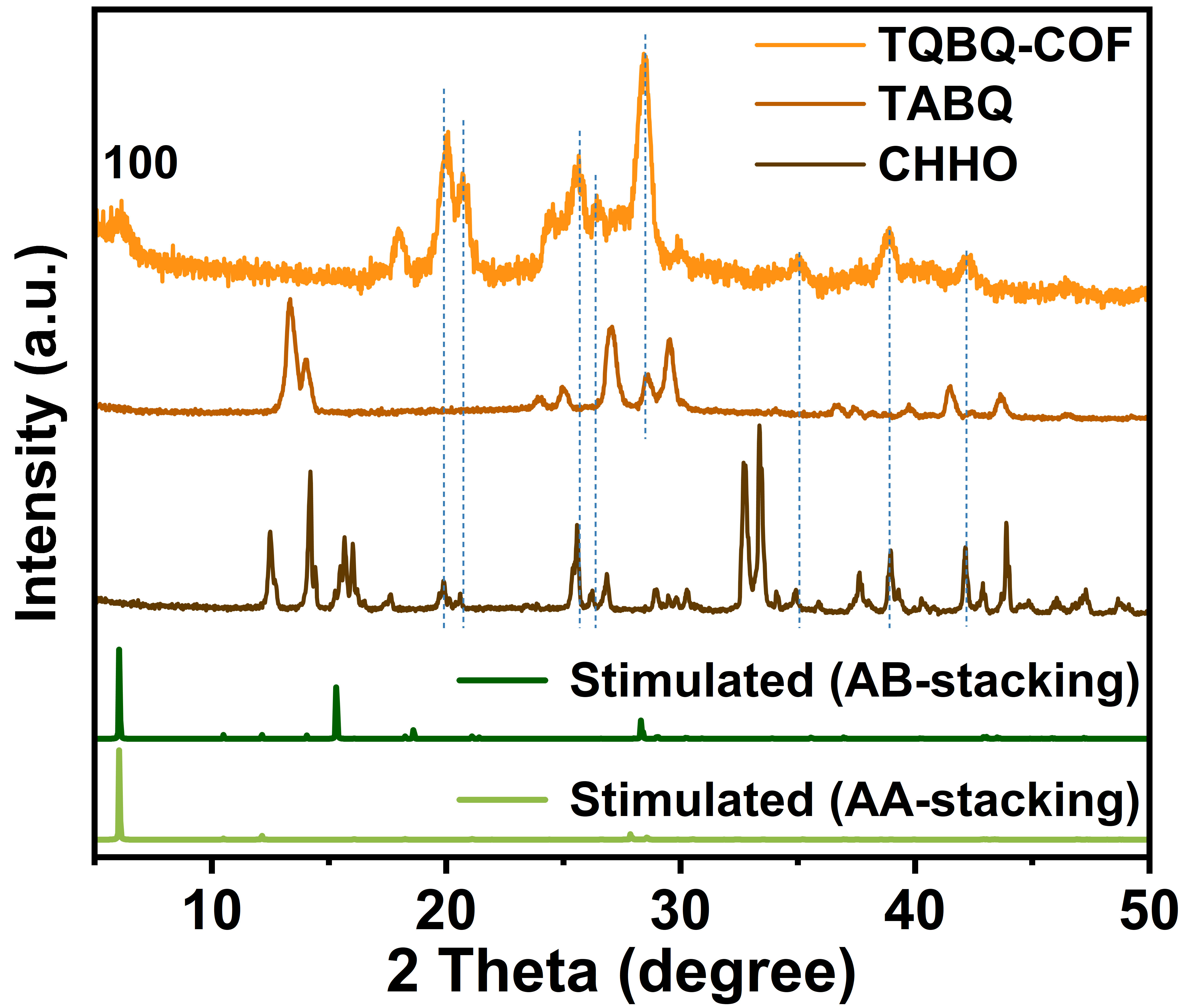
**

**Figure S3** XRD patterns of monomer TABQ, CHHO and TQBQ-COF (initial COF).


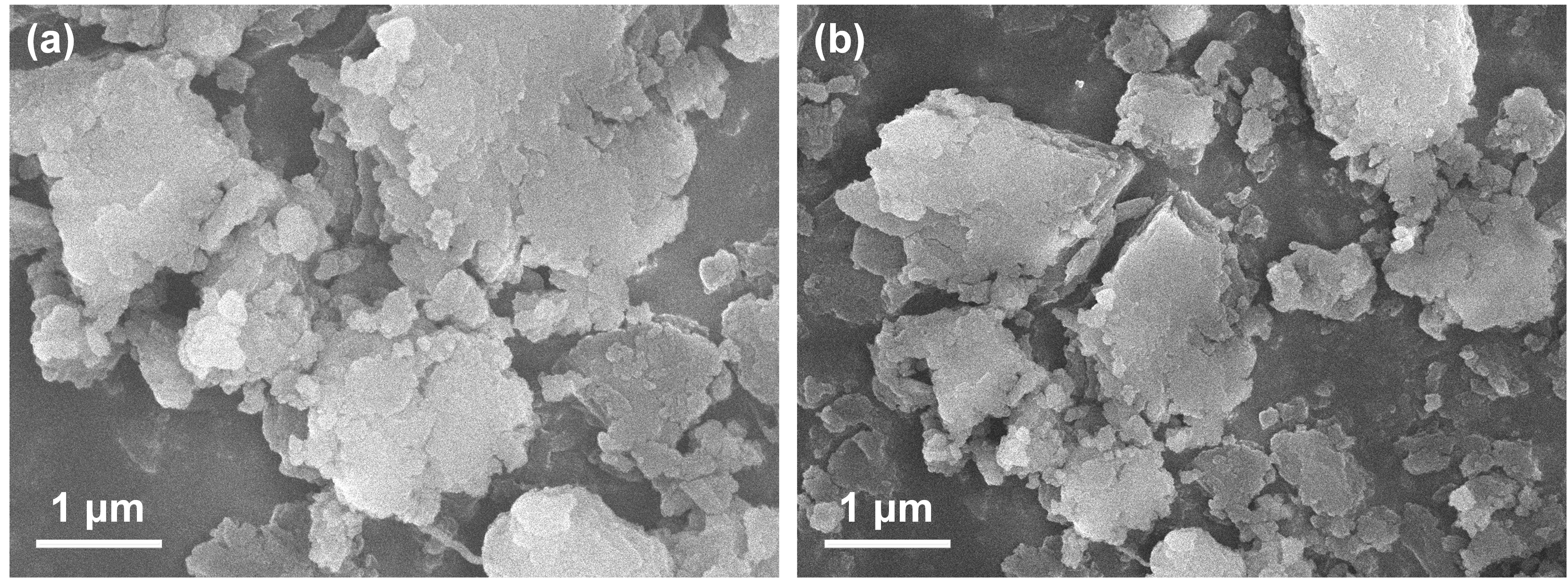


**Figure S4** SEM images of (a) initial COF and (b) ED-COF.


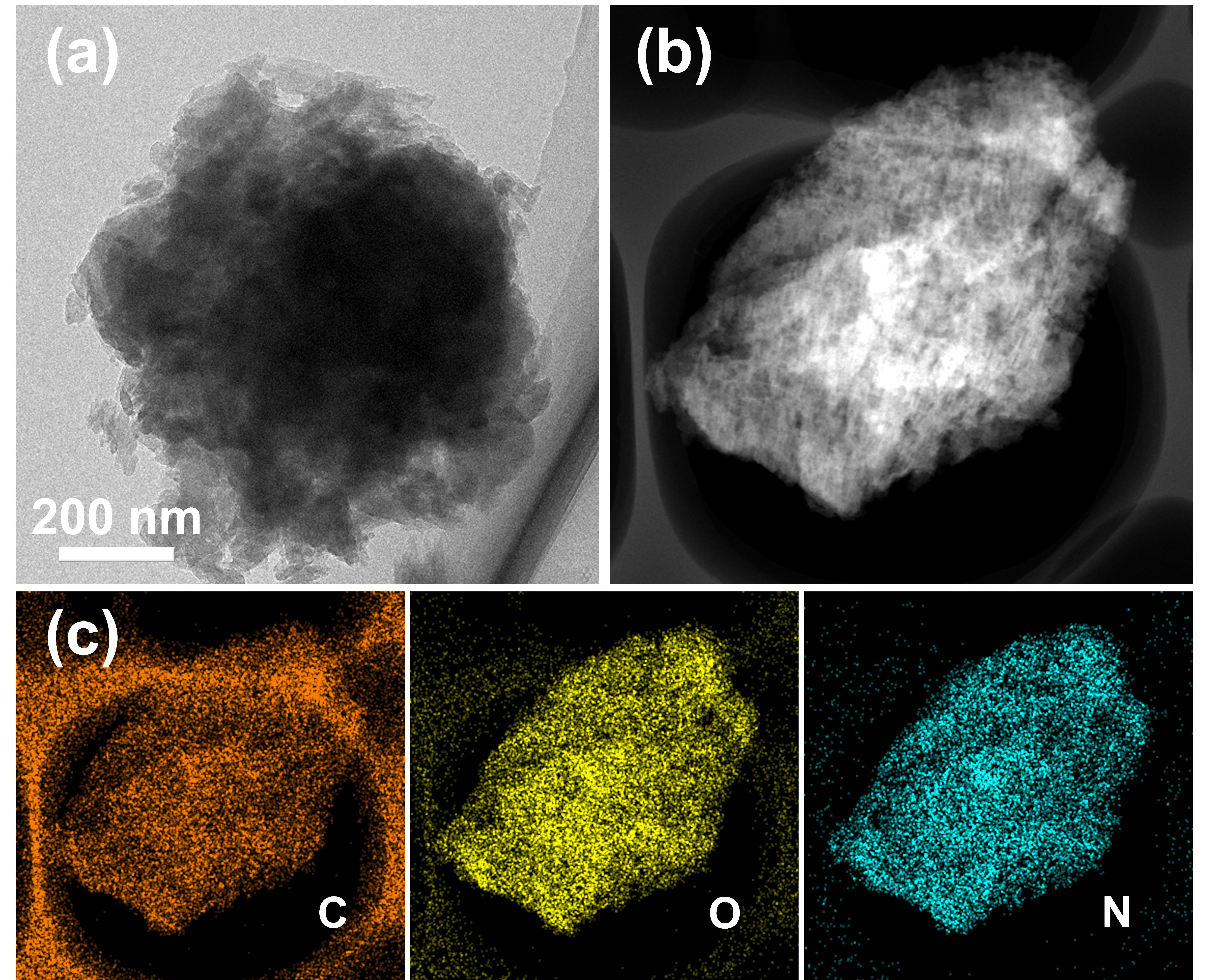


**Figure S5** (a) TEM image, (b) HAADF and (c) EDS mapping images of TQBQ-COF (initial COF).


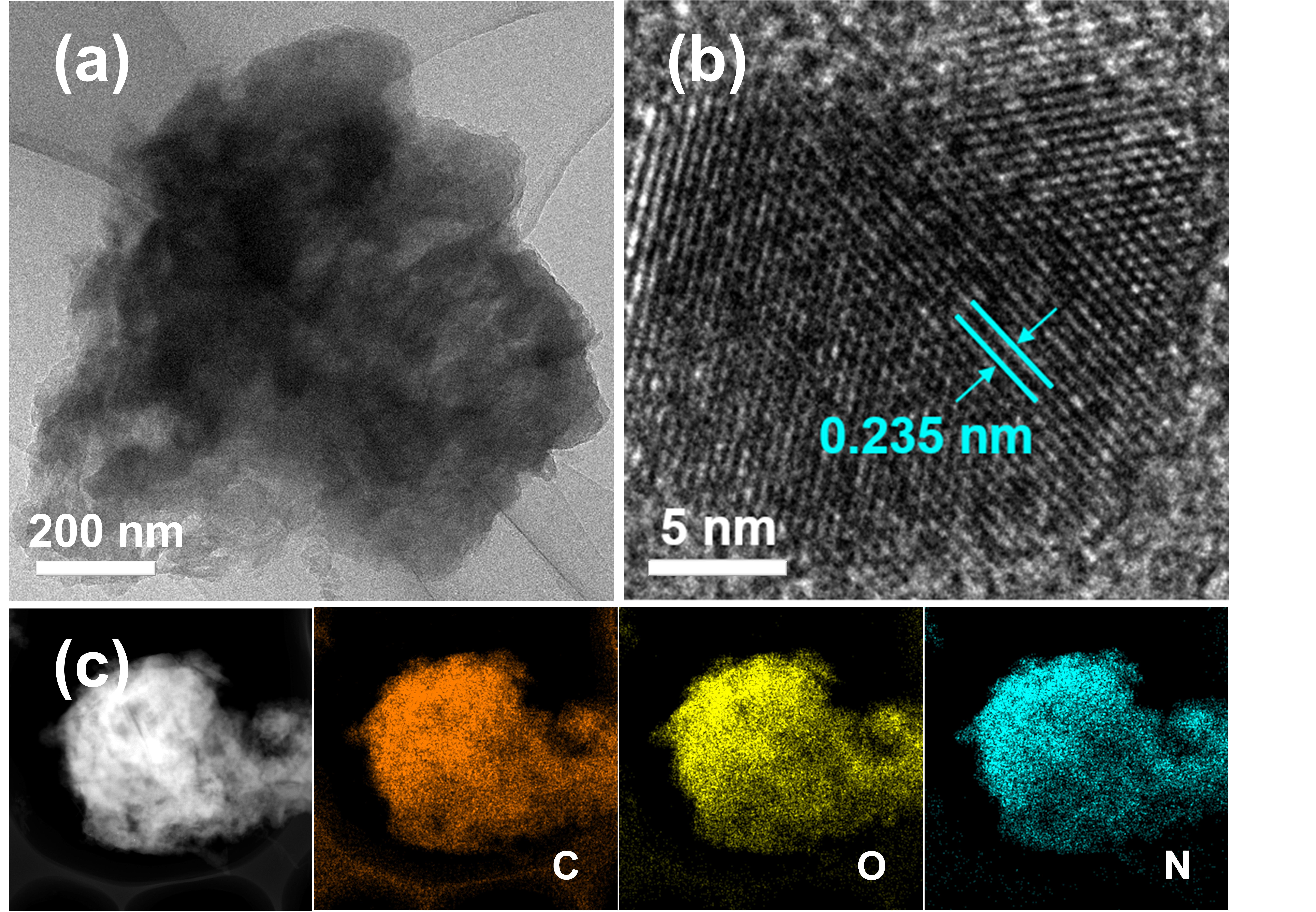


**Figure S6** (a) TEM image, (b) HRTEM image and (c) HAADF and EDS mapping images of ED-COF.


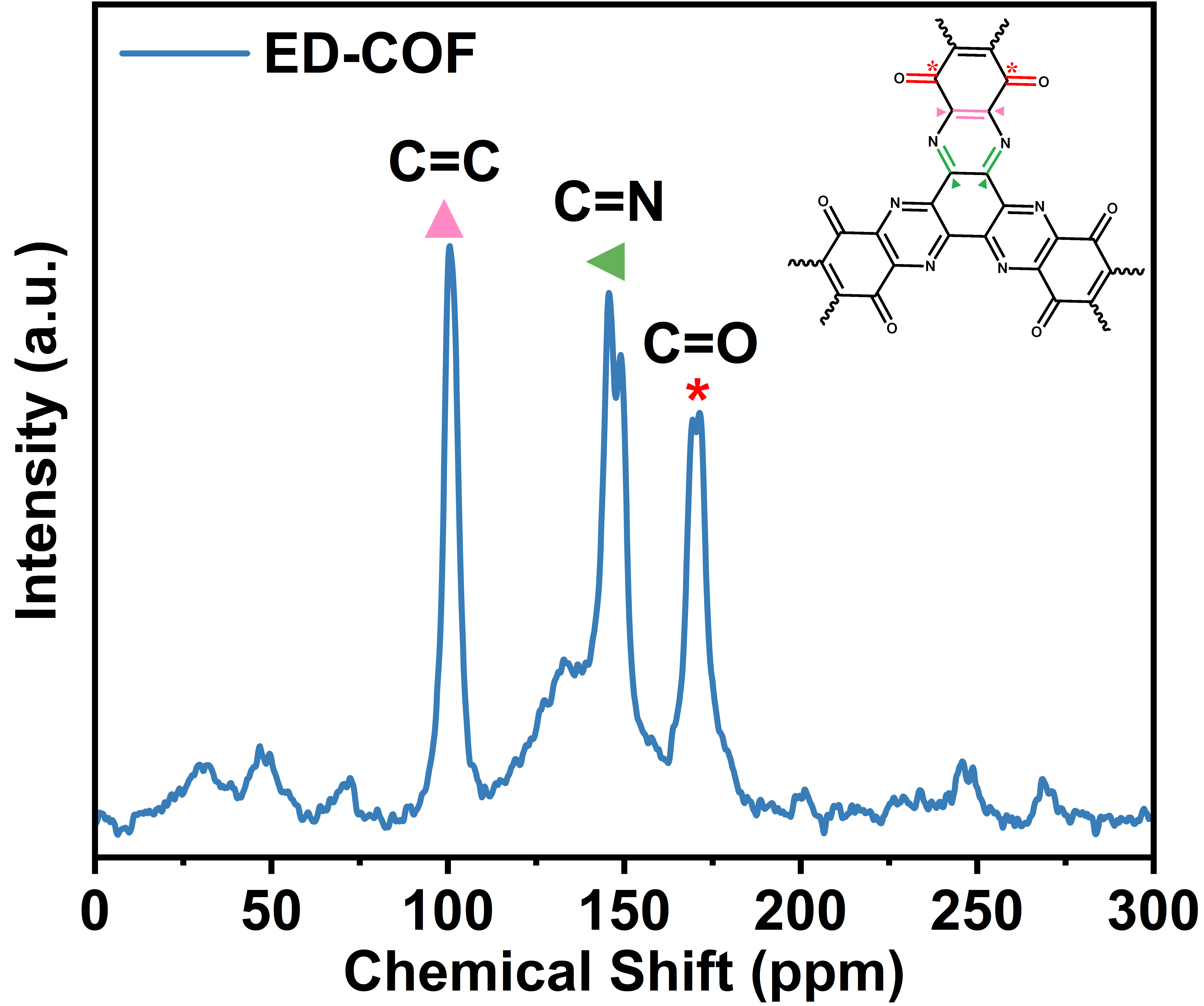


**Figure S7** Solid-state ^13^C NMR spectrum of ED-COF (inset: main functional groups of ED-COF).

**
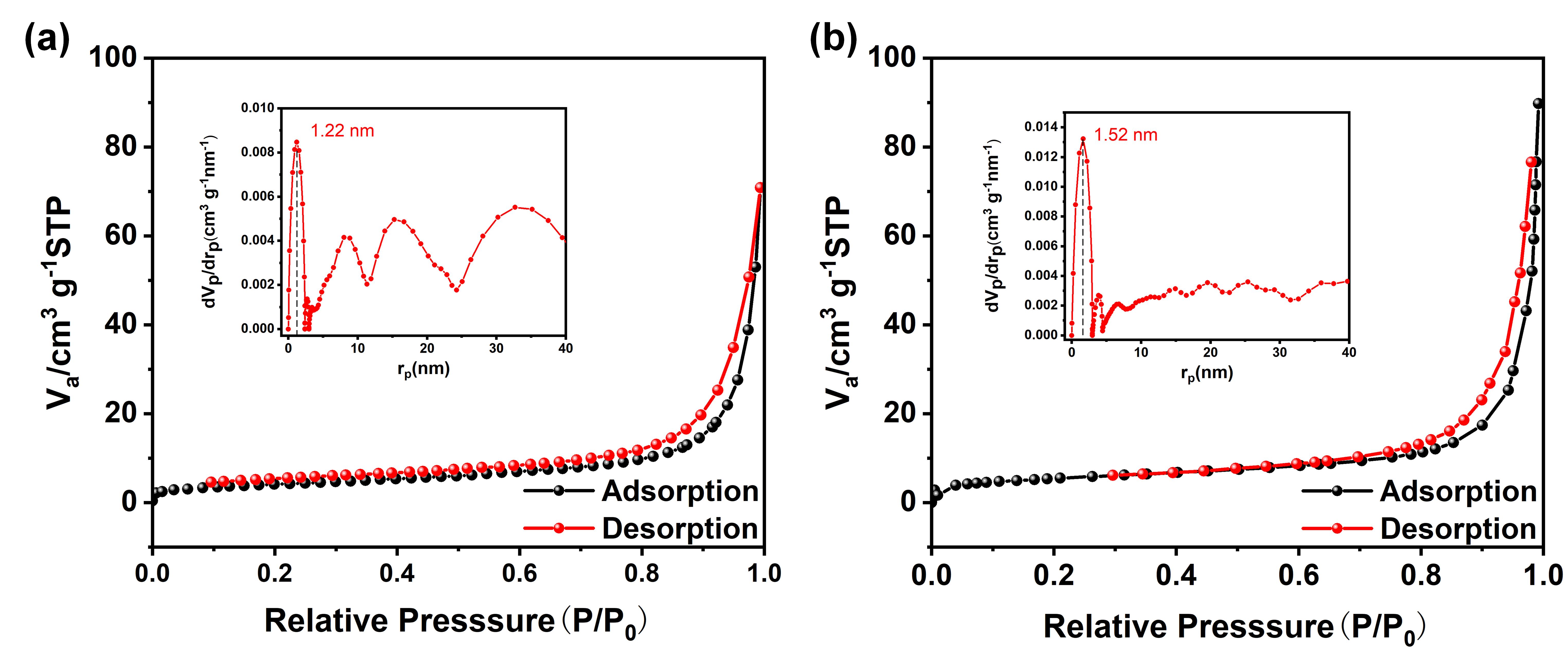
**

**Figure S8** (a) N_2_ adsorption-desorption isotherm and pore size distribution curve (inset) of initial COF; (b) N_2_ adsorption-desorption isotherm and pore size distribution curve (inset) of ED-COF.


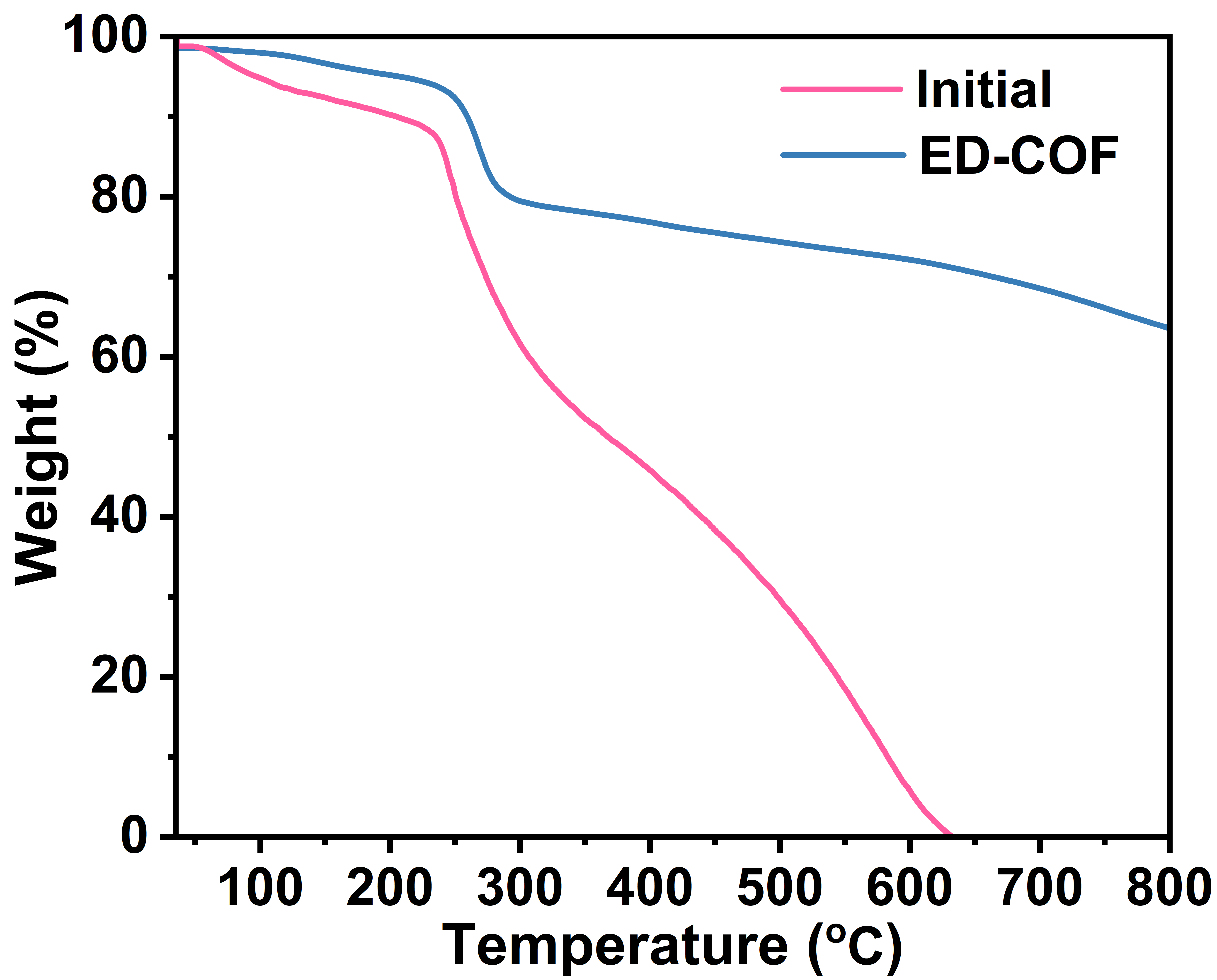


**Figure S9** TGA curves of initial COF and ED-COF.

**
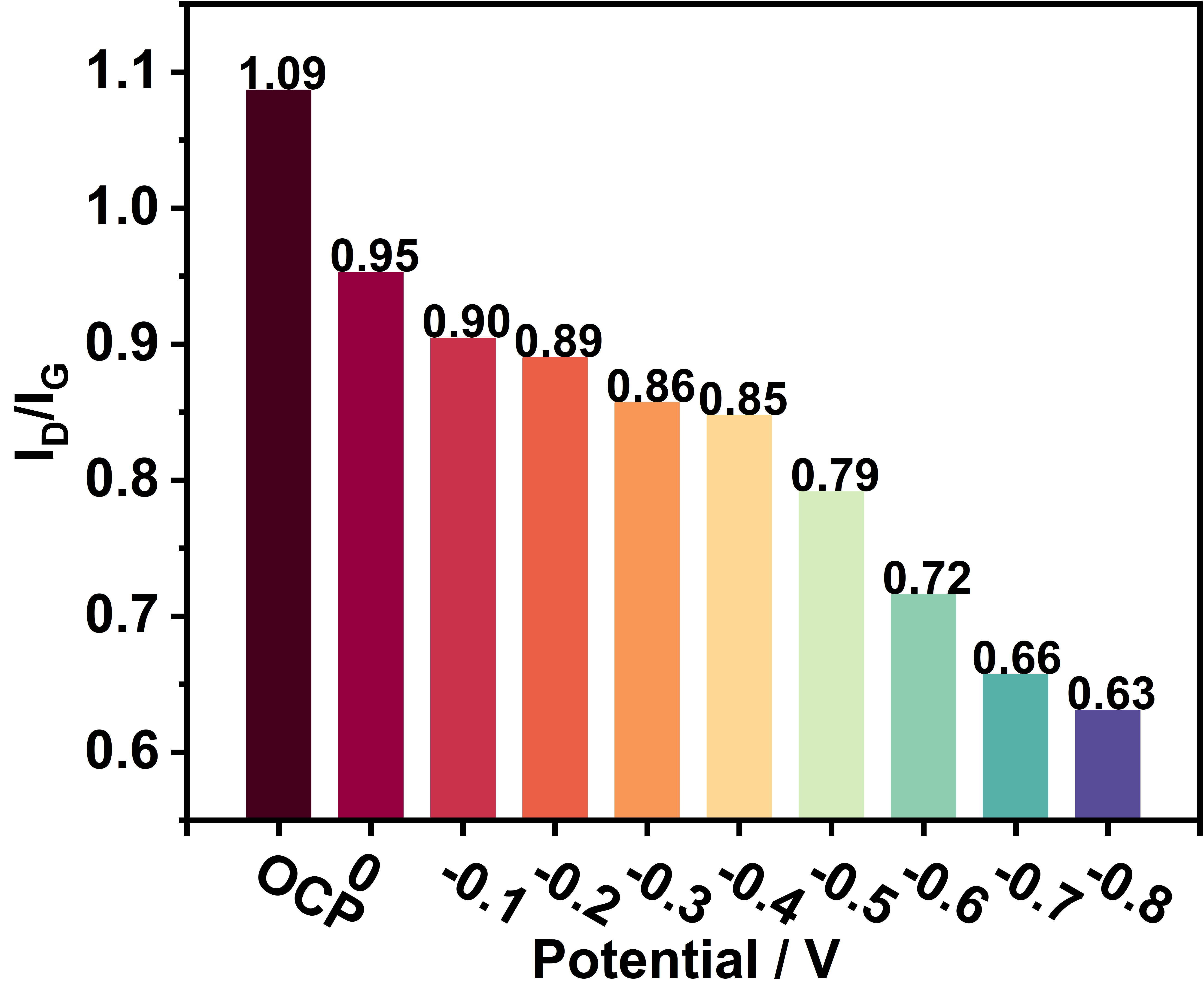
**

**Figure S10** I_G_/I_D_ of TQBQ-COF (initial COF) at various potentials.

**
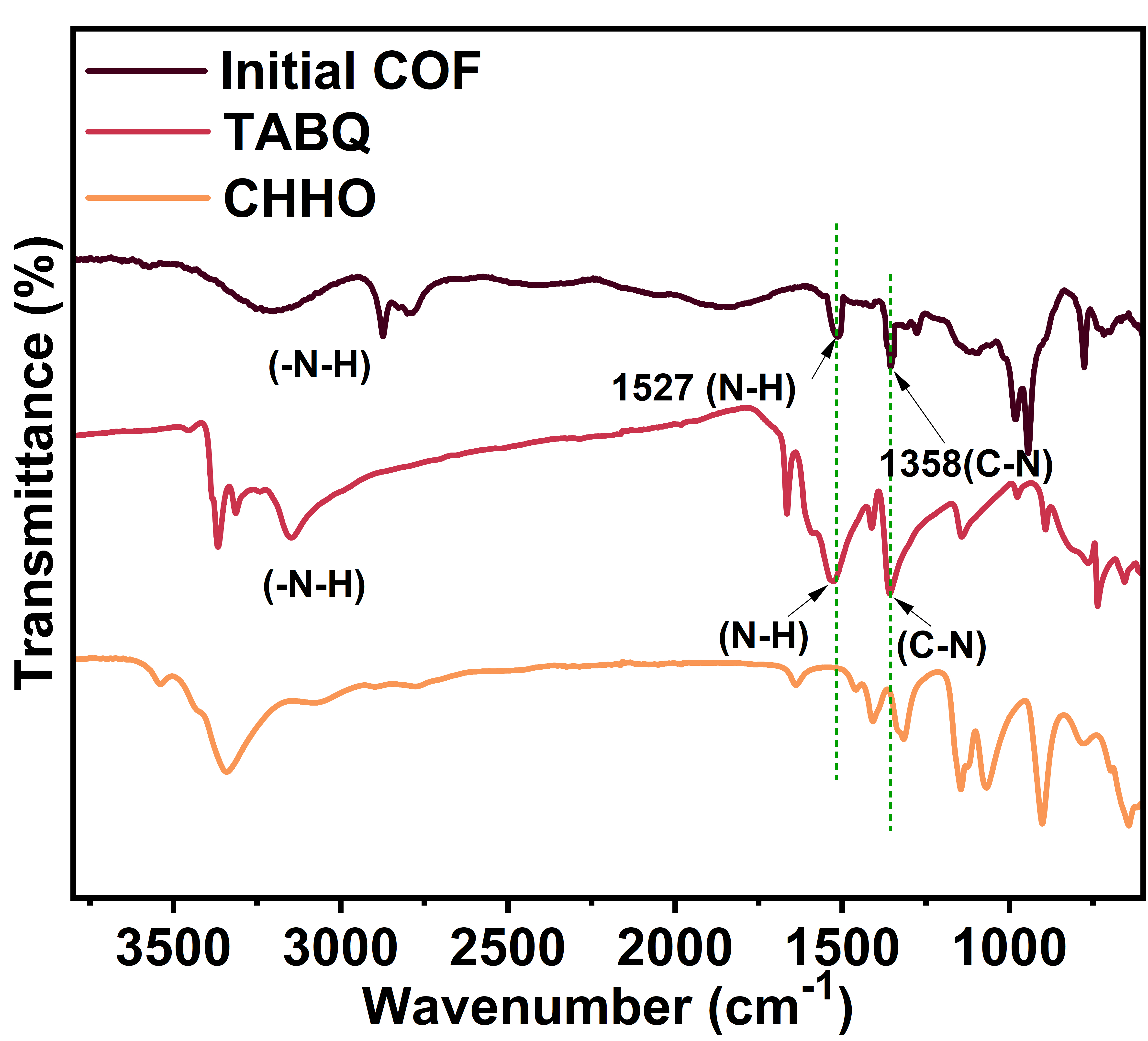
**

**Figure S11** FT-IR spectra of monomer TABQ, CHHO and TQBQ-COF (initial COF).


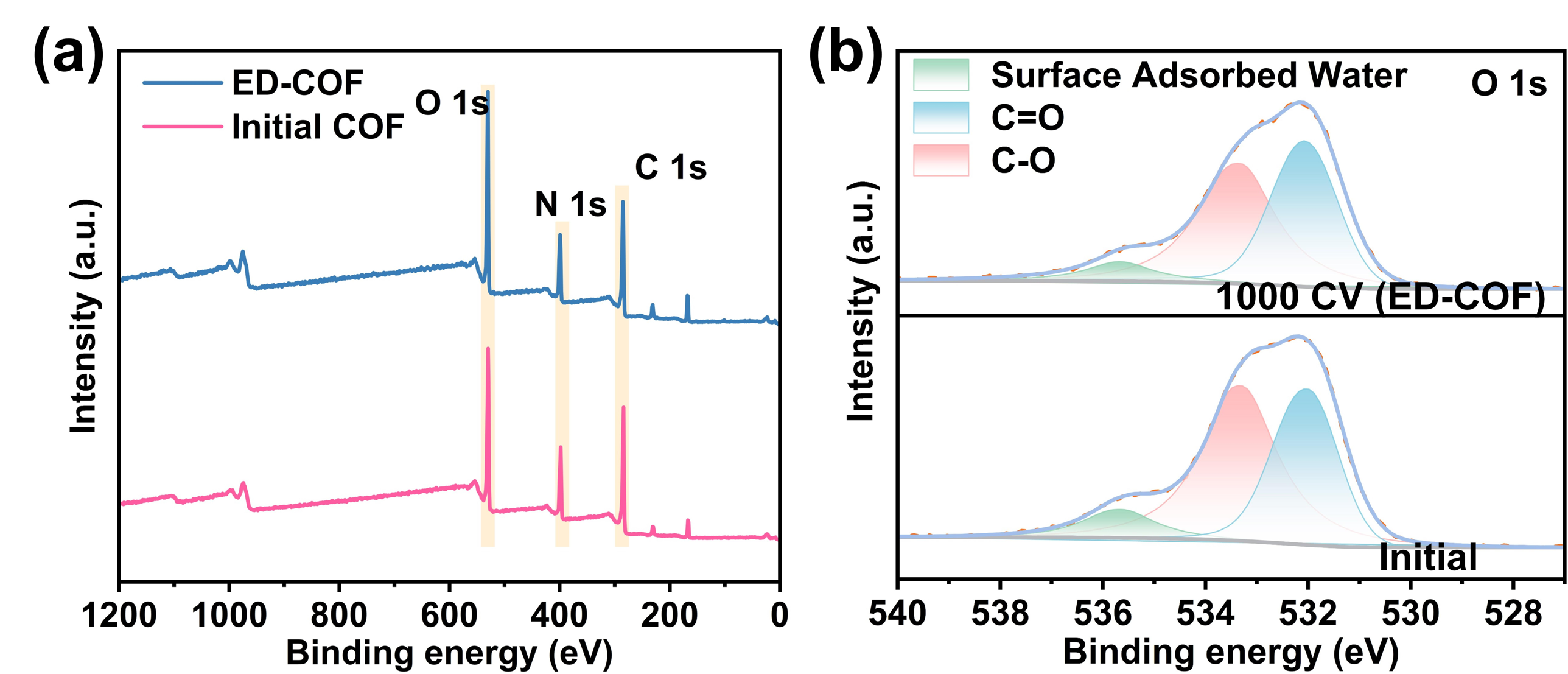


**Figure S12** (a) XPS survey spectra and (b) O 1s spectra of initial COF and ED-COF.

**
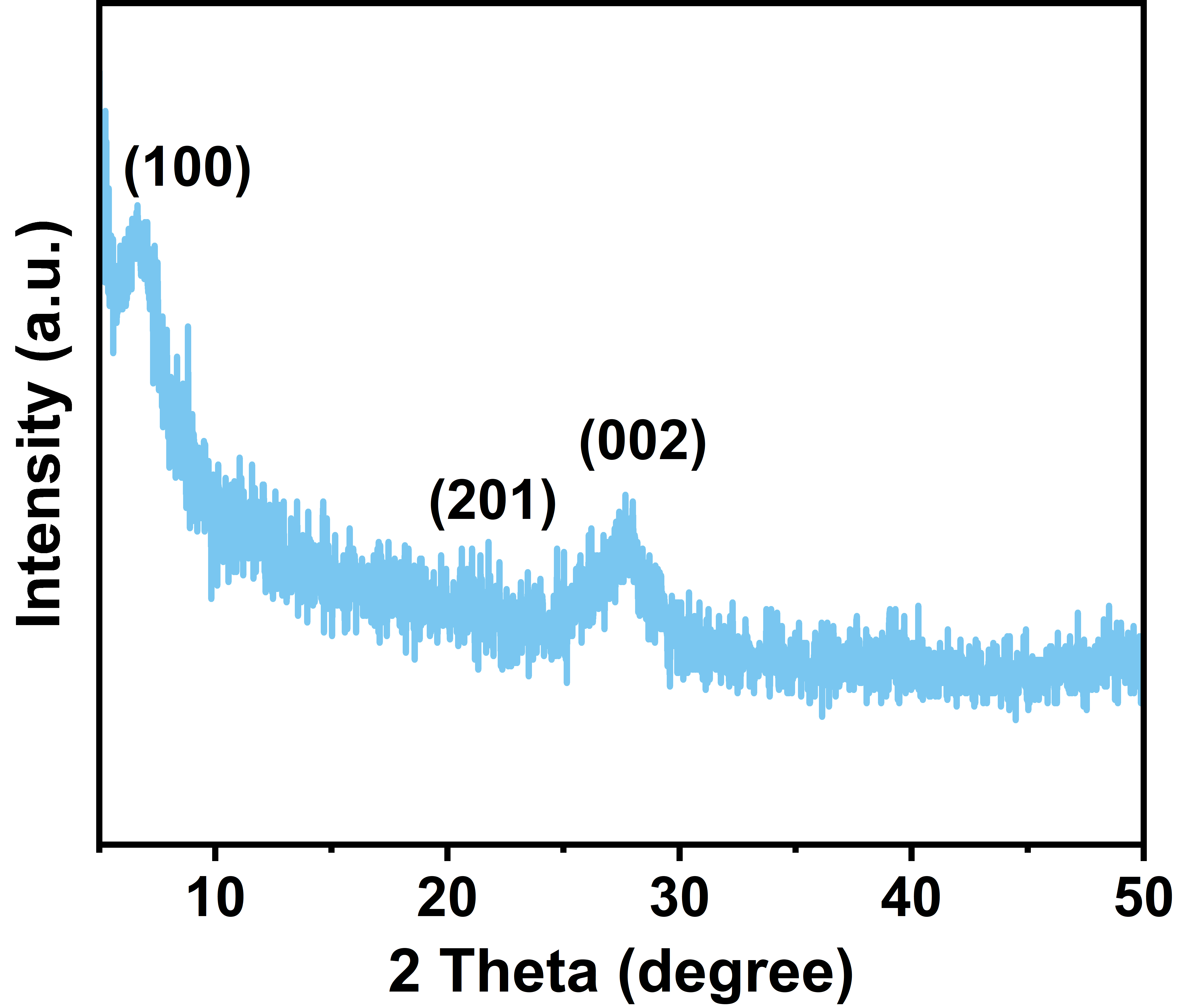
**

**Figure S13** XRD pattern of ED-COF after stability test.

**

**

**Figure S14** TEM image of ED-COF after stability test.





**Figure S15** (a) XPS survey spectrum, (b) C 1s, (c) N 1s, (d) O 1s of ED-COF after stability test.


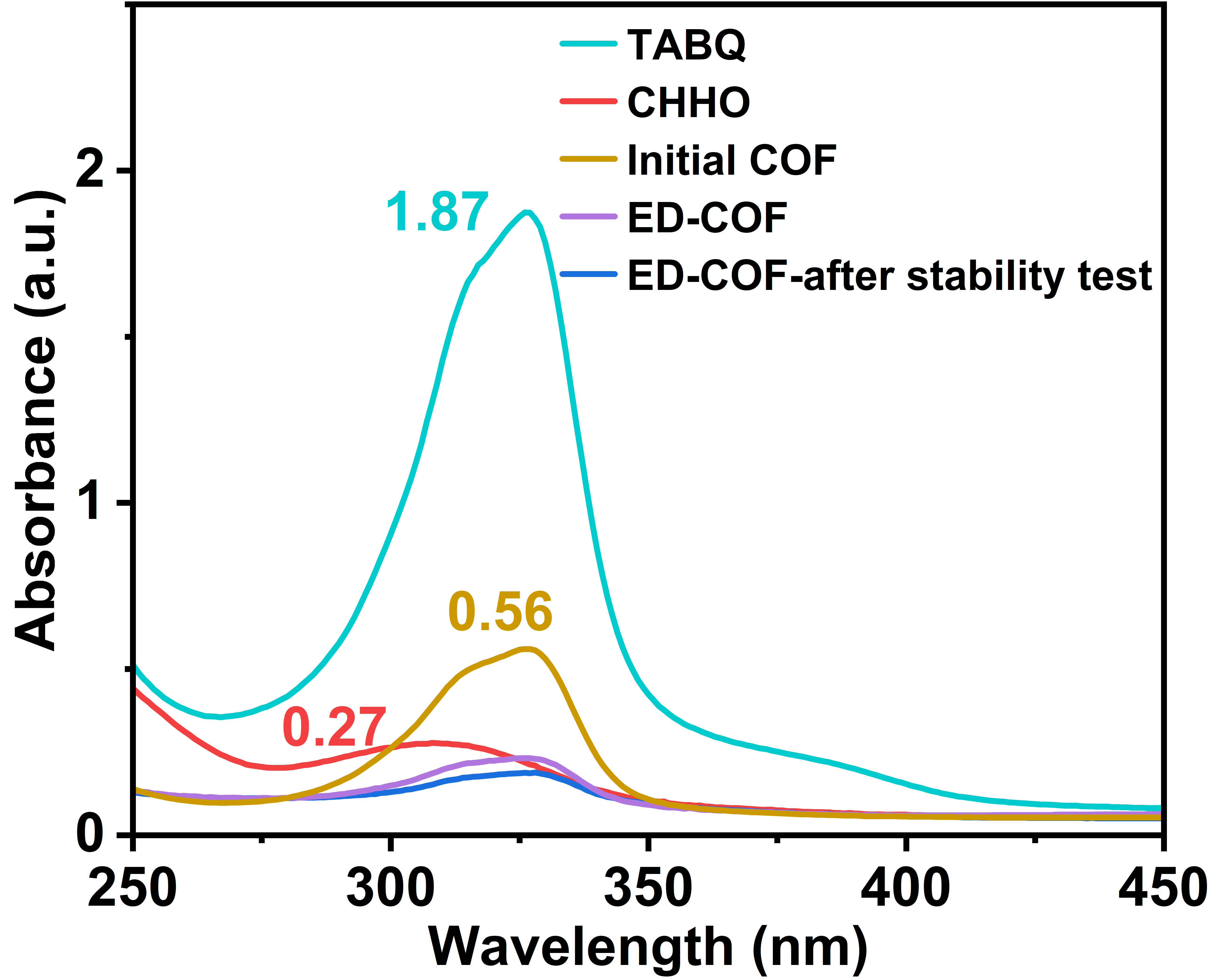


**Figure S16.** The UV-Vis absorption spectrum of these samples.

Monomer TABQ, monomer CHHO, initial COF and ED-COF were separately dispersed in 0.5 M H_2_SO_4_. Moreover, the electrolyte after long-term stability test of ED-COF was also collected. Ultraviolet-visible (UV-Vis) absorption spectroscopy tests were performed on these electrolytes. As shown in Figure S15, the UV-Vis absorption spectrum of monomer TABQ exhibits a significant absorption peak at 327 nm, with an absorbance of 1.87, indicating that TABQ has good solubility in 0.5 M H_2_SO_4_ and generates a chromophore with strong absorption characteristics in the UV region. In contrast, monomer CHHO shows only a weak absorption peak at 310 nm with an absorbance of 0.27, suggesting its poor solubility in 0.5 M H_2_SO_4_. For initial COF, the absorption peak position is consistent with that of TABQ, and the absorbance is 0.56, which is mainly attributed to the residual unreacted TABQ dissolved in0.5 M H_2_SO_4_, thus exhibiting a certain level of absorbance. It is noteworthy that both ED-COF and its post-stability-test counterpart exhibit extremely low absorbance, which indicates that ED-COF does not undergo significant dissolution in 0.5 M H_2_SO_4_, demonstrating excellent chemical stability.

**

**

**Figure S17** (a) C 1s, (c) N 1s, (c) O 1s XPS spectra of Benzoxazole linkage COF.





**Figure S18** (a) C 1s, (c) N 1s, (c) O 1s XPS spectra of Benzimidazole linkage COF.

**

**

**Figure S19** (a) C 1s, (c) N 1s, (c) O 1s XPS spectra of β-ketoeneamine linkage COF.

**Table S1** Comparison of HER performance of ED-COF with the reported COF-based catalysts in acid media

| Electrocatalysts | Electrolyte | Overpotential @ 10 mA cm^−2^ (mV) | Tafel slope (mV dec^-1^) | Reference |
| --- | --- | --- | --- | --- |
| **ED-COF** | **0.5 M H_2_SO_4_** | **103.6** | **70.3** | **This work** |
| TQBQ-COF (initial) | 0.5 M H_2_SO_4_ | 169.4 | 179.5 | This work |
| SB-PORPy-COF | 0.5 M H_2_SO_4_ | 380 @ 5 mA cm^−2^ | 116 | [4] |
| TpPAM-COF | 0.5 M H_2_SO_4_ | 250 | 106 | [5] |
| 2DCCOF1 | 0.5 M H_2_SO_4_ | 541 | 130 | [6] |
| BPY-CTF | 0.5 M H_2_SO_4_ | 345 | 94.3 | [7] |
| DCP-CTF | 0.5 M H_2_SO_4_ | 155 | 53.4 | [7] |
| BPT-COF | 0.5 M H_2_SO_4_ | 142 | 110 | [8] |
| Ru@COF | 1.5 M H_2_SO_4_ | 212 | 79 | [9] |
| Ru@COF-1 | 0.5 M H_2_SO_4_ | 200 | 140 | [10] |
| PETA-PAM COF | 0.5 M H_2_SO_4_ | 261 | 122 | [11] |
| JLNU-COFs-301 | 0.5 M H_2_SO_4_ | 189 | 85.4 | [12] |
| JLNU-COFs-302 | 0.5 M H_2_SO_4_ | 151 | 101.6 | [12] |

**Reference**

1. P.-F. Wei, M.-Z. Qi, Z.-P. Wang, S.-Y. Ding, W. Yu, Q. Liu, L.-K. Wang, H.-Z. Wang, W.-K. An, W. Wang, *J. Am. Chem. Soc.* **2018**, *140*, 4623-4631.
2. Das, S. K. Mandal, *Chem. Mater.* **2019**, *31*, 1584-1596.
3. S. Chandra, D. Roy Chowdhury, M. Addicoat, T. Heine, A. Paul, R. Banerjee, *Chem. Mater.* **2017**, *29*, 2074-2080.
4. S. Bhunia, S. K. Das, R. Jana, S. C. Peter, S. Bhattacharya, M. Addicoat, A. Bhaumik, A. Pradhan, *ACS Appl. Mater. Interfaces* **2017**, *9*, 23843-23851.
5. B. C. Patra, S. Khilari, R. N. Manna, S. Mondal, D. Pradhan, A. Pradhan, A. Bhaumik, *ACS Catal.* **2017**, *7*, 6120-6127.
6. D. Zhou, X. Tan, H. Wu, L. Tian, M. Li, *Angew. Chem. Int. Ed.* **2019**, *58*, 1376-1381.
7. B. Zhang, Y. Zhang, M. Hou, W. Wang, S. Hu, W. Cen, X. Cao, S. Qiao, B.-H. Han, *J. Mater. Chem. A* **2021**, *9*, 10146-10159.
8. Y. Bai, Y. Liu, M. Liu, X. Wang, S. Shang, W. Gao, C. Du, Y. Qiao, J. Chen, J. Dong, Y. Liu, *Angew. Chem. Int. Ed.* **2022**, *61*, e202113067.
9. S. Maiti, A. R. Chowdhury, A. K. Das, *ChemNanoMa*t. **2020**, *6*, 99-106.
10. Y. Zhao, Y. Liang, D. Wu, H. Tian, T. Xia, W. Wang, W. Xie, X.-M. Hu, X. Tian, Q. Chen, *Small* **2022**, *18*, 2107750.
11. S. Samanta, S. Khatun, I. Mukherjee, S. Maity, M. A. Addicoat, A. Pradhan, *Sustain. Energy Fuels* **2024**, *8*, 4183-4191.
12. Y. Ma, Y. Fu, W. Jiang, Y. Wu, C. Liu, G. Che, Q. Fang, *J. Mater. Chem. A* **2022**, *10*, 10092-10097.
